# Operative Times, Costs and Patient‐Related Outcome Measures in Vertical Ridge Augmentation With Customised Reinforced PTFE Mesh Versus CAD/CAM Titanium Mesh: Secondary Analysis of a Randomised Clinical Trial

**DOI:** 10.1111/jcpe.14185

**Published:** 2025-05-26

**Authors:** Alessandro Cucchi, Sofia Bettini, Lucia Tedeschi, Debora Franceschi, Istvan Urban, Antonino Fiorino, Giuseppe Corinaldesi

**Affiliations:** ^1^ Private Practice Bologna Italy; ^2^ Department of Experimental and Clinic Medicine University of Florence Florence Italy; ^3^ Department of Periodontology and Oral Medicine University of Michigan Ann Arbor Michigan USA; ^4^ Department of Neuroscience and Reproductive and Odontostomatological Sciences, ‘Federico II’ University of Naples Naples Italy; ^5^ Department of Biomedical and Neuromotor Sciences (DIBINEM) University of Bologna Bologna Italy

**Keywords:** alveolar ridge augmentation, customised mesh, guided bone regeneration, patient‐reported outcome measure

## Abstract

**Aim:**

This secondary analysis of a randomised clinical trial aimed to investigate vertical ridge augmentation (VRA) by comparing complication rates (primary outcome), times, costs and patient‐reported outcome measures (PROMs) between customised Ti‐reinforced PTFE mesh and customised CAD/CAM titanium mesh.

**Materials and Methods:**

Patients with vertical bone defects were randomly assigned to alveolar bone augmentation using either Ti‐PTFE mesh or Ti mesh (T0). After 6–9 months, barriers were removed, and computer‐guided surgery was performed to place implants in the augmented sites (T1). Complications, times, costs and PROMs (anxiety, pain, anti‐inflammatory drug dosage, limitations in daily functions) were assessed and analysed.

**Results:**

Forty‐eight of 50 patients completed the bone augmentation surgery as per protocol. The estimated difference in healing complications was −0.04% (CI: −0.22 to 0.13), confirming the non‐inferiority of Ti meshes to PTFE meshes. The estimated differences were −3.50 min (CI: −23.49 to 16.49) for total operative time (*p* = 0.688); €17.37 (−77.25 to 111.99) for costs (*p* = 0.130); −0.17 (CI: −0.80 to 0.47) for anti‐inflammatory drug usage (*p* = 0.299); 0.56 (CI: −1.97 to 0.85) for pain levels (*p* = 0.565); and −0.13 (CI: −0.61 to 0.36) for limitations in daily functions (*p* = 0.528), on the day after surgery.

**Conclusion:**

Outcomes were favourable, which encourage the use of both medical devices with low complication rates and both digital approaches, resulting in favourable operative times and PROMs.

## 1 Introduction

Guided bone regeneration (GBR) is a widely used technique for vertical ridge augmentation (VRA) despite its technical difficulty and invasiveness, leading to anxiety, stress and discomfort for both clinicians and patients (Jepsen et al. 2019; Poli et al. 2022; Shi et al. 2023).

Recent technological advances, such as digital planning and CAD/CAM, have improved operative efficiency, reduced operative time and reduced post‐operative discomfort and pain, albeit with increase in overall costs (Urban et al. 2023; Zhou et al. 2022).

Among the digital approaches for VRA, customised CAD/CAM titanium meshes and customised PTFE meshes have been investigated, reporting their effectiveness, strengths and benefits (Chiapasco et al. 2021; Cucchi et al. 2021, 2022; Lizio et al. 2022; Hartmann et al. 2022; Felice et al. 2024). Sofar, however, only a few authors (Felice et al. 2024) have considered patient‐related outcome measures (PROMs) in randomised clinical studies.

Given the increasing emphasis on treatment efficiency, cost effectiveness, and patient‐centred care in contemporary implant dentistry, we considered it clinically and scientifically relevant to perform a secondary analysis of a previous randomised clinical trial on VRA with a customised reinforced PTFE mesh versus a CAD/CAM titanium mesh. The present analysis aimed to compare healing complication rates (primary outcome) as well as operative times, costs and PROMs (secondary outcomes) in order to investigate the invasiveness, costs and durations of the two procedures in a more comprehensive evaluation from a clinician‐ and patient‐oriented perspective. Information regarding surgical and healing complications, vertical bone gain, regeneration rates and volumetric changes has been published by us previously (Cucchi et al. 2024a, 2024b).

## 2 Materials and Methods

### 2.1 Study Design

The present study was designed as a non‐inferiority, parallel‐group, double‐blind, randomised clinical trial. This report comprises the secondary analysis of a previously published article (Cucchi et al. 2024a, 2024b) and was prepared following the CONSORT guidelines (Schulz et al. 2014) and adhering to the criteria set by the Implant Dentistry‐Core Outcome Set and Measurement (ID‐COSM) initiative as described by Tonetti et al. (2023).

The trial was conducted according to the ethical principles set forth in the Declaration of Helsinki (2014). The study protocol (protocol CMF 01/2019) obtained ethics approval from the Central‐Emilia‐Wide‐Area Ethical Committee of the Emilia‐Romagna Region (CE‐AVEC, study‐code ce19143), and the protocol was registered on ClinicalTrials.gov (NCT04257097).

The inclusion and exclusion criteria were described in a previous pilot study reporting preliminary results (Cucchi et al. 2022). Briefly, healthy adult patients showing a vertical bone defect (VBD) of > 3 mm and up to five teeth, requiring three‐dimensional (3D) bone augmentation for prosthetically guided implant placement, were enrolled and treated between 2020 and 2022.

The patients were randomly assigned to two study groups, according to a computer‐generated simple randomization, which was known only to the second surgeon (S.B.). One‐half of the patients were treated with a Ti‐reinforced PTFE mesh covered with a collagen membrane (‘control group’ or ‘PTFE group’), while the other half was treated with a customised titanium mesh covered with a collagen membrane (‘test group’ or ‘Ti‐mesh group’).

The patient, first surgeon, examiner and statistician were blinded to the treatment allocation. All the patients were treated by the same first surgeon (A.C.), who had experience with both PTFE membranes and titanium meshes. The first surgeon (A.C.) was blinded until the second surgeon (S.B.), serving as surgical assistant, opened the randomisation envelope and revealed the allocation group for each patient, just before barrier device filling and fixation.

### 2.2 Surgical Protocol

All surgical procedures have already been carefully described in a previous publication related to the same research protocol (Cucchi et al. 2024a, 2024b).

#### 2.2.1 Digital Planning and Device Manufacturing

**FIGURE 1:**
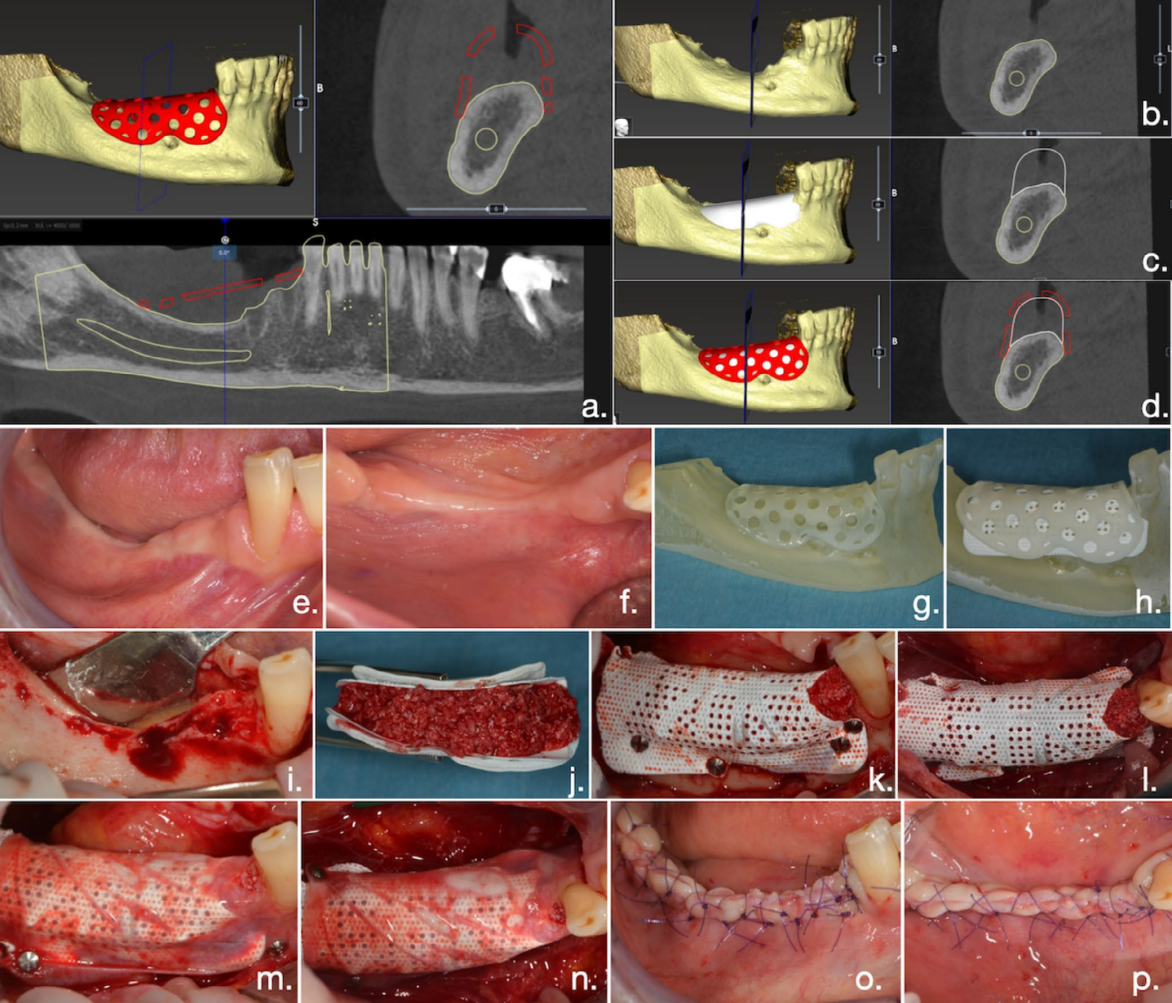
Clinical case belonging to the PTFE group. (a) Representation of virtual bone augmentation process for control group (PTFE group), carried out on FICOM files extracted from pre‐operative CBCT. (b) Initial bone defect. (c) Virtual bone augmentation. (d) Virtually planned customised mesh. (e, f) Pre‐operative intraoral clinical view. (g) Augmented 3D bone model and replica of the mesh. (h) PTFE mesh modelled and tailored, placed between the 3D bone model and the replica. (i) Clinical view of the bone defect. (j) Pre‐modelled PTFE mesh, supported by the replica and filled with the graft mixture. (k, l) Filled PTFE mesh placed and fixed in the surgical site. (m, n) PTFE mesh covered with a resorbable collagen membrane. (o, p) Tension‐free primary closure obtained with a combination of horizontal mattress sutures and multiple interrupted sutures.

**FIGURE 2:**
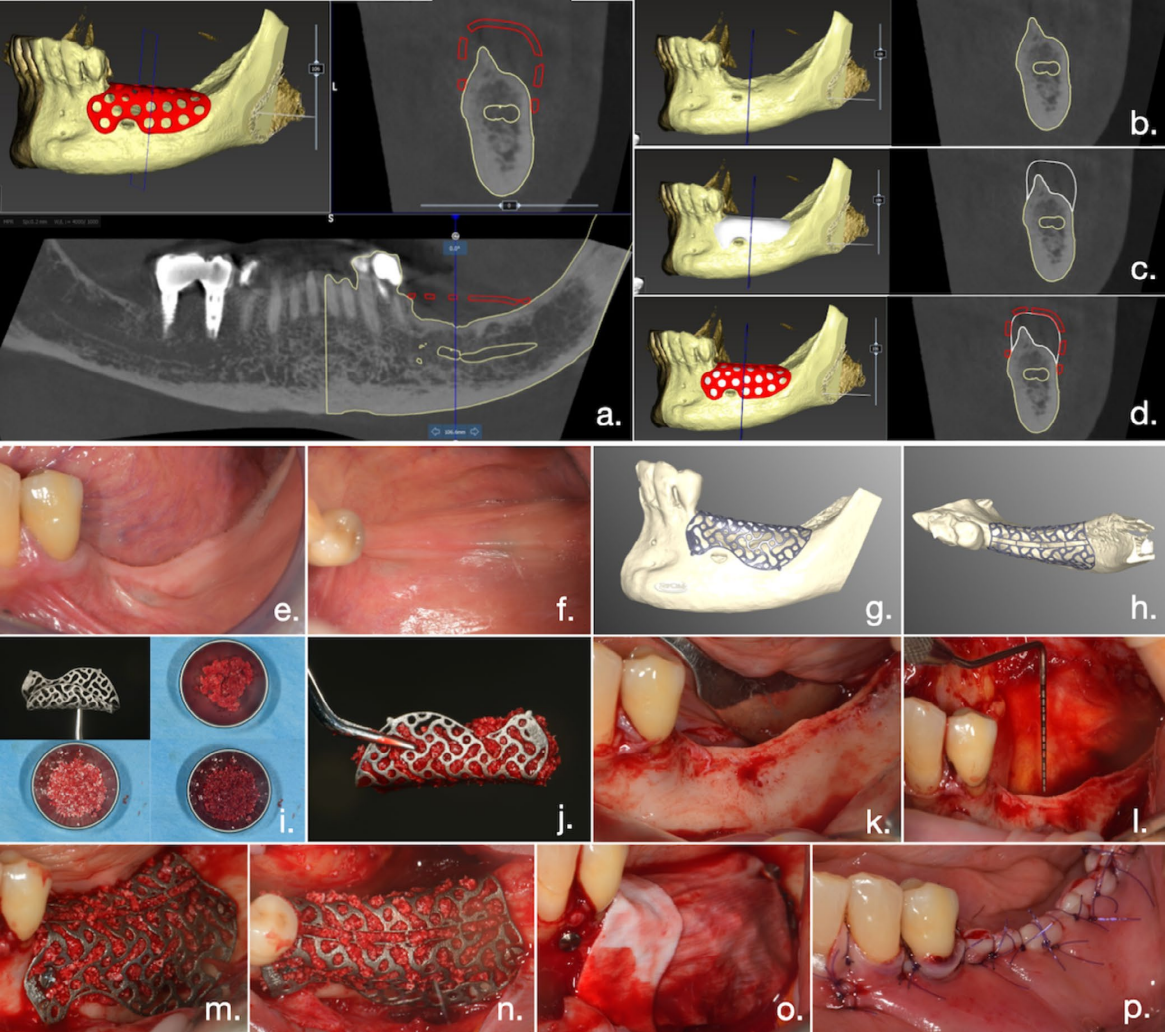
Clinical case belonging to the Ti mesh group. (a) Representation of virtual bone augmentation process for control group (PTFE group), carried out on FICOM files extracted from pre‐operative CBCT. (b) Initial bone defect. (c) Virtual bone augmentation. (d) Virtually planned customised mesh. (e, f) Pre‐operative intraoral clinical view. (g, h) Virtual rendering of customised titanium mesh. (i) Customised titanium mesh and mixture of autogenous bone, xenograft and peripheral venous blood. (j) Customised titanium mesh filled with the graft mixture. (l) Mobilisation of lingual flap. (k) Clinical view of the bone defect. (m, n) Customised titanium mesh fixed in the surgical site. (o) Customised titanium mesh covered with a resorbable collagen membrane. (p) Tension‐free primary closure obtained with a combination of mattress sutures and multiple interrupted sutures.

A blinded clinician (G.C.) with knowledge and experience in implantology and bone augmentation planned all the virtual bone augmentations of all defects, working and prototyping on STL files on a dedicated software (OPERA, BTK, Biotec, Italy). Then, either the 3D bone model and the replica of the mesh project for the PTFE group (Figure 1a–d) or the CAD‐CAM Ti mesh project for the T‐mesh group (Figure 2a–d) were approved by the second surgeon (S.B.) and produced by the manufacturers.

#### 2.2.2 Bone Augmentation Surgery

At time 0 (T0), the patients underwent premedication with antibiotic and anti‐inflammatory drugs 1 h prior to surgery. Immediately before surgery, patients underwent a thorough oral rinsing protocol with (i) povidone iodine, (ii) hydrogen peroxide and (iii) chlorhexidine (0.20%); then, a local anaesthetic and sedatives were administered (2 mg/2 mL delorazepam and 10 mg/2 mL diazepam).

After incision and full‐thickness elevation, the surgical flaps were mobilised properly with a single superficial periosteal incision on the buccal side (Ronda and Stacchi 2015; Urban et al. 2016) and with a smooth incision of the mylohyoid muscle associated with a periosteal incision (in anterior area) on the lingual side (Urban et al. 2018); in the maxilla, Bichat's buccal fat pad technique was adopted if necessary (Cucchi et al. 2023). Autogenous bone was harvested and mixed in a 50:50 ratio with deproteinised bovine bone mineral (DBBM) (Bio‐oss, Geistlich) and hydrated with peripheral venous blood. The actual allocation was disclosed to the first surgeon only at this stage.

The digital approach used in the control group has already been carefully described (Cucchi et al. 2022): Briefly, the pre‐modelled PTFE mesh (RPM, Osteogenics), supported by the replica of the mesh, was filled with the graft mixture, placed in the surgical site and fixed using titanium osteosynthesis screws (Pro‐Fix System, Osteogenics) and/or tacks (membrane fixation system, MCBio); a resorbable membrane (Vitala, Osteogenics) was placed over the PTFE mesh and stabilised with 2–3 titanium tacks (Figure 1e–p).

In the test group, the Ti mesh (YxossCBR, Reoss) was similarly filled with the same graft mixture, placed in the surgical site and stabilised using osteosynthesis screws. The mesh was covered with a resorbable membrane (Biogide, Geitslich) and fixed with tacks (Figure 2e–p).

Finally, the augmented sites were covered with tension‐free flaps and closed with a double layer of 5/0 sutures (ResorbaGlycolon, Osteogenics). After surgery, the patients received a diary form to be filled out daily with all required information in detail.

#### 2.2.3 Implant Surgery

**FIGURE 3:**
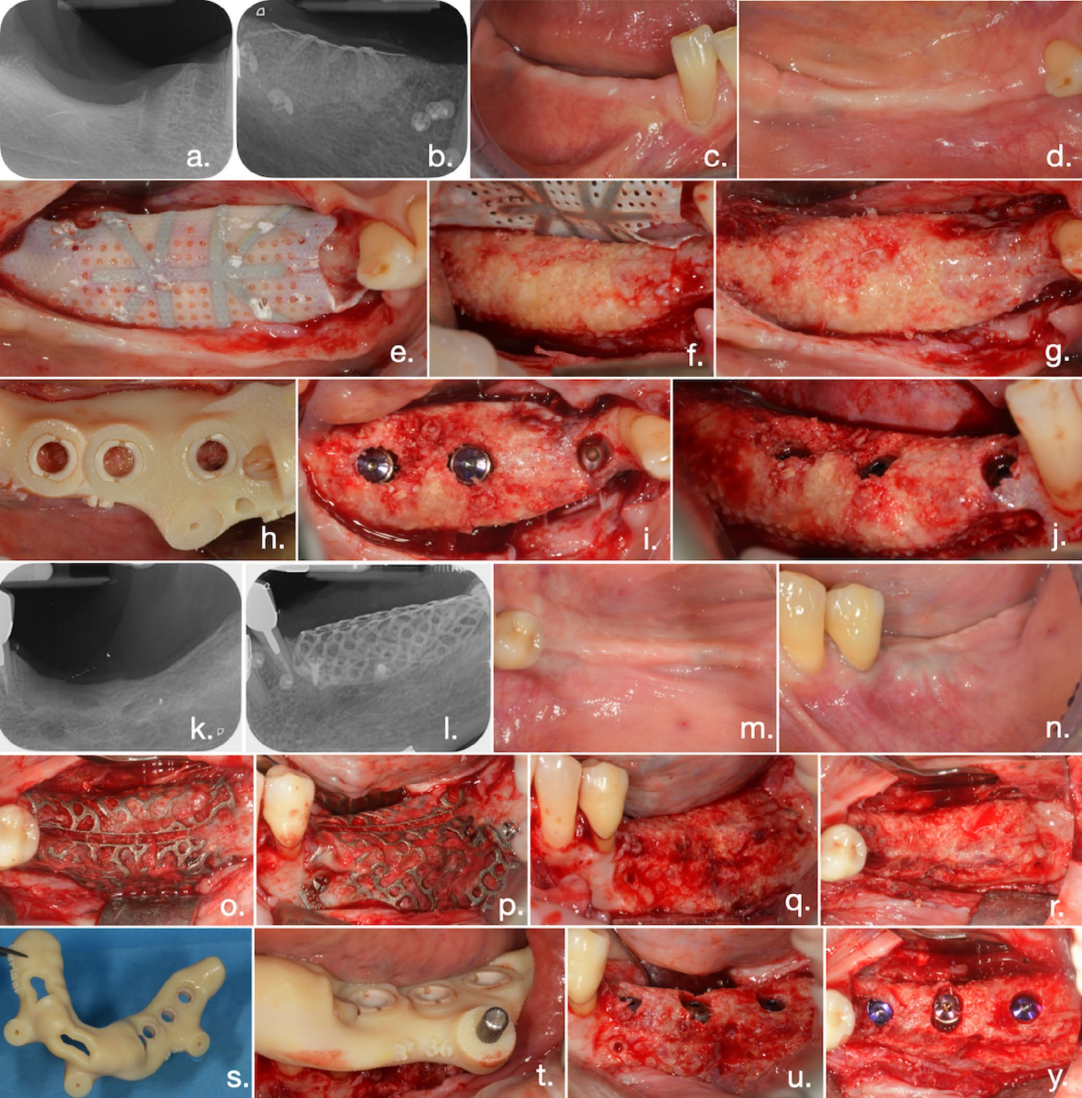
Re‐entry surgery of clinical case belonging to the PTFE group: (a) Pre‐operative intraoral radiograph. (b) Post‐operative endoral radiograph. (c, d) Intraoral clinical view before re‐opening. (e) Re‐opening of the treated area. Exposure of the RPM, (f, g) Removal of the RPM, clinical view of regenerated bone. (h) Implants site preparation and implants placement using surgical guide template. (i, j) Implants placed in regenerated bone. Re‐entry surgery of clinical case belonging to Ti mesh group: (k) Pre‐operative intraoral radiograph. (l) Post‐operative endoral radiograph. (m, n) Intra‐oral clinical view before reopening. (o, p) Re‐opening of the treated area: Exposure of the Ti mesh; (q, r) Removal of the titanium mesh: Clinical view of regenerated bone; (s, t) Surgical guide template used for implant placement. (u–y) Implants placed in regenerated bone.

At time 1 (T1) (6 months for mandible and 9 months for the upper maxilla), a second CBCT and intraoral scanning were performed, and implant placement was virtually planned using a dedicated software (Navimax, Biomax) for a static computer‐assisted approach. During the surgery, the barrier device was removed, along with all osteosynthesis screws and tacks. The implant sites were prepared using a surgical guide, and planned tapered implants (T3 Implants, Zimvie) were placed in the prosthetically driven position and axis using the same surgical guide (Figure 3).

#### 2.2.4 Prosthetic Restoration

Finally, at time 2 (T2), the implants were restored with screw‐retained fixed crowns or bridges and followed up after functional loading.

### 2.3 Data Collection

All clinical data were collected by a blinded examiner (L.T.), using a specific case report form. The population data had already been recorded and analysed (Cucchi et al. 2024a, 2024b).

The operative times for each VRA surgery were recorded and divided as follows:

– Pre‐operative times: digital virtual planning; manufacturing of customised device; modelling PTFE mesh; total pre‐operative time.
– Intra‐operative times: durations of each surgical phase: site preparation, flap mobilisation, bone harvesting, mesh fixation, membrane fixation, suturing; actual intra‐operative time (sum of each single phase); total intra‐operative time (from incision to complete suture, including downtime); and the total chairside time.
– Total patient chairside time: amount of time on the dental chair (from admission to discharge).

The costs associated with VRA surgery were analysed based on the defect size, categorised as small (1 tooth), medium (2–3 teeth) and large (more than 3 teeth).

In particular, the following costs associated with VRA surgery were recorded: shipping expenses; anaesthesia, based on the number of vials used; stereolithographic resin model and the replica of the device; bone scraper; barrier device (PTFE mesh or Ti mesh) and resorbable membrane based on the defect size; biomaterial (DBBM); mini‐screws and/or tacks, based on the quantity used; and sutures, based on the quantity used.

The PROMs were evaluated as follows:

1. Level of anxiety, as recorded on a 100‐mm visual analogue scale (VAS) ranging from 0 (‘no anxiety’) to 100 (‘worst anxiety imaginable’) immediately before and immediately after surgery (Skowron et al. 2017);
2. Treatment satisfaction associated with quality of life, as entered on the Oral‐Health‐Impact‐Profile‐14 (OHIP‐14) questionnaire, immediately before (Campos et al. 2021) and 6 months after surgery (Jenei et al. 2015);
3. Level of discomfort, as recorded on a post‐operative symptom severity (PoSSe) scale filled out 14 days after surgery (Ruta et al. 2000);
4. Level of pain, self‐reported by each patient in a medication diary, as recorded on a 100‐mm visual analogue scale (VAS) from day 0 to day 14;
5. Dosage of anti‐inflammatory drugs (i.e., number of 600 mg ibuprofen tablets), self‐reported by each patient in a medication diary, taken from the day of surgery to 14 days after surgery;
6. Limitations in daily functions (swallowing, breathing, eating, speaking, mouth‐opening and other ongoing daily activities), self‐reported by each patient in a medication diary, as recorded on a 5‐point scale, from day 0 to day 14;
7. Incidence of post‐operative symptoms (swelling, nausea, bruising, haemorrhage, fever, reduction in skin sensitivity and difficulty opening the mouth), self‐reported by each patient in a medication diary, reported as dichotomous variables (yes/no), on days 1, 3, 7 and 14 and recorded by means of photographs of the patient taken by himself/herself (selfies) and/or by relatives (portraits);
8. Willingness to undergo the same type of surgery, recorded at days 7 and 14 on a 4‐point scale ranging from 1 (‘I would have no problem undergoing this type of surgery again if needed’) to 4 (‘I would never undergo this type of surgery again’).

Clinician‐reported outcomes measures (ClinRO), such as level of anxiety, level of stress and level of satisfaction, were assessed and analysed as reported in Appendix S1.

### 2.4 Statistical Analysis

All data were entered by a single blinded operator (A.F.). Prior to entry, all data were evaluated for accuracy and completeness. Healing complication rates were considered as primary outcomes, while operative times, costs, PROMs and ClinROs were the secondary outcomes.

The analysis, carried out at the patient level (statistical unit of analysis), was based on the hypothesis (H1) that the incidence of healing complications (primary outcome) in the Ti mesh (test) group would not be inferior to those in the PTFE mesh (control) group. The non‐inferiority test was performed for complications only (one‐sided 95% confidence interval approach) and justified a sample size of 50 participants based on the following parameters: significance level (α) of 0.05; power (1 – *β*) of 0.8; expected proportion of 0.041 (4.1%) in the control group; expected proportion of 0.14 (14%) in the treatment group; and a margin of non‐inferiority set at 0.1. The mean values for the control group were derived from the last relevant review (Urban et al. 2019).

A non‐inferiority design was chosen because it would be more appropriate in settings where there is an established effective treatment (PTFE membranes or meshes, as previously published), and the goal is to show that the new treatment (customised Ti meshes) is not significantly worse, especially when it could offer other benefits (lower complication, lower economic cost, shorter operative time, more favourable PROMs, etc.). Detailed reasons are described and explained in Appendix S2.

In addition, superiority analyses were conducted to evaluate significant differences in terms of the intervention group and all other variables. To ensure that the observed effects were due to the treatment itself rather than external factors, and to reduce the risk of bias due to non‐compliance or protocol deviations, a per‐protocol analysis was performed. More information about the statistical analysis is reported in Appendix S2.

## 3 Results

### 3.1 Per‐Protocol Analysis

In total, 50 out of 58 screened patients with a mean age of 56 years (range: 30–70 years; 14 males and 36 females) were enrolled and underwent the surgery. Forty‐eight patients completed the bone augmentation surgery as planned (two patients experienced flap perforation) and were available for the per‐protocol analysis. The study was initially designed as a multi‐centre trial involving a university centre and a private centre; however, because of the COVID‐19 pandemic, the university centre was unable to enrol any patients, leading to all 50 patients being recruited and treated exclusively at the private centre. No dropouts occurred in this phase. The primary and secondary outcomes are reported below.

#### 3.1.1 Complication Rates

**FIGURE 4:**
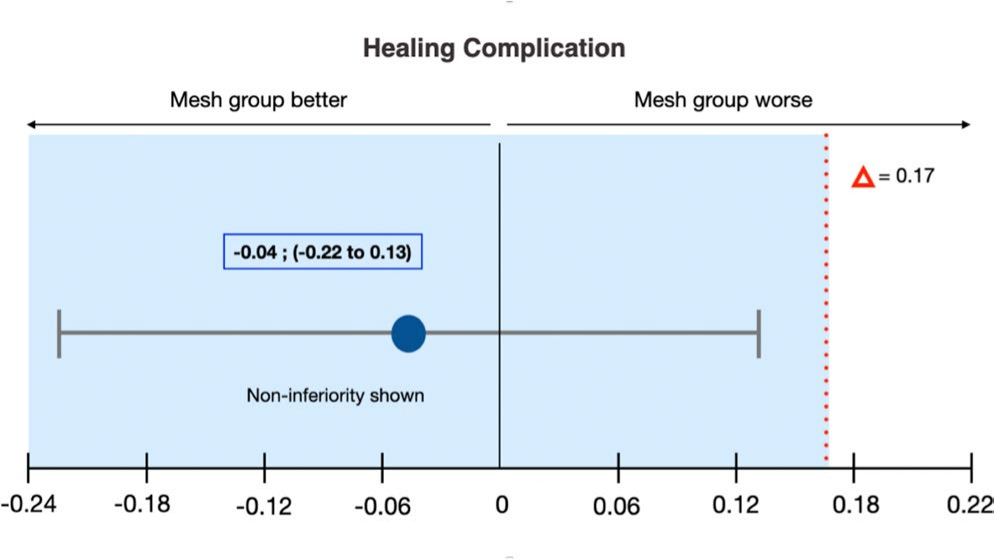
Error bars indicating one‐sided 95% confidence intervals of the difference in healing complication mean values between the test and control groups (mesh minus PTFE). The broken red line indicating the difference in the score (= 0.17) shows the non‐inferiority margin (delta). The shaded area indicates the zone of non‐inferiority. The CI does not include Δ and the data prove non‐inferiority of the Ti mesh group to the PTFE group. Although there is no statistically significant difference between the two treatments, the test group tends to be better than the control group in terms of healing complications.

Between T0 and T1, five healing complications occurred (three in PTFE group and two in the Ti mesh group): three were classified as early complications (within first 3 months) and two as late complications (after the third month). Non‐inferiority analysis of healing complications showed that the Ti mesh group was non‐inferior to the PTFE group (Figure 4). The estimated difference in complication rates was −0.04 (95% CI: −0.22 to 0.13), with the non‐inferiority margin of 0.1 respected, confirming the non‐inferiority of the Ti meshes to the PTFE meshes. Additionally, no statistically significant differences were observed between the two groups (PTFE 12.5% vs. Ti mesh 8.3%) (*p* = 0.645). Data related to the study population are reported in Appendix S3, while other non‐inferiority analyses and univariate regression analyses related to complications are reported in Appendix S4.

#### 3.1.2 Operative Times

The digital virtual planning of bone augmentation required a mean time of 16.4 ± 6.6 min and 18.6 ± 8.0 min, respectively (*p* = 0.304). Ti meshes require additional time for uploading to a dedicated platform and interfacing with the engineers via email, so the total time for planning came to 84.4 ± 15.5 min (*p* < 0.0001). It is important to note that PTFE mesh required additional time for modelling the device using a replica (average time of 10.2 ± 7.4 min) just before surgery. The total pre‐operative times, excluding the shipping times, were 26.6 ± 11.9 and 84.4 ± 15.5 min in the two study groups, respectively (*p* < 0.0001).

The effective intra‐operative times in the two study groups were 96.3 ± 19.1 and 102.2 ± 27.1 min (*p* = 0.386), respectively. Regarding intra‐operative data, the average total surgical time was 121.7 ± 37.2 min for the PTFE group and 118.2 ± 31.3 min for the Ti mesh group, with no statistically significant differences (*p* < 0.0001). The total operative times, including both pre‐operative and intra‐operative times, were 148.3 ± 41.0 and 221.1 ± 30.0 min in the two study groups, respectively (*p* < 0.0001). Regarding the surgical phases, only the fixation of resorbable membranes over the meshes showed a significant difference of 3.83 min (CI: 1.87–5.7925) (*p* = 0.0003).

All data related to durations of each pre‐operative and intra‐operative phase, including the total chairside times, and univariate regression analysis are shown in Appendix S4.

#### 3.1.3 Costs

The total cost of the VRA procedure averaged €1200 ± 177.4 for the PTFE group and €1272.4 ± 148.3 for the Ti mesh group, without statistically significant differences (*p* = 0.132). The specific costs of each material needed to perform the VRA surgery and univariate regression analysis are shown in Appendix S4. The cost of screws and tacks showed significant differences of €26.25 (CI: 7.58–44.92) (*p* = 0.0106) and −€28.58 (CI: −45.80 to −11.37) (*p* = 0.0017), respectively.

#### 3.1.4 Patient‐Reported Outcome Measures (PROMs)

**FIGURE 5:**
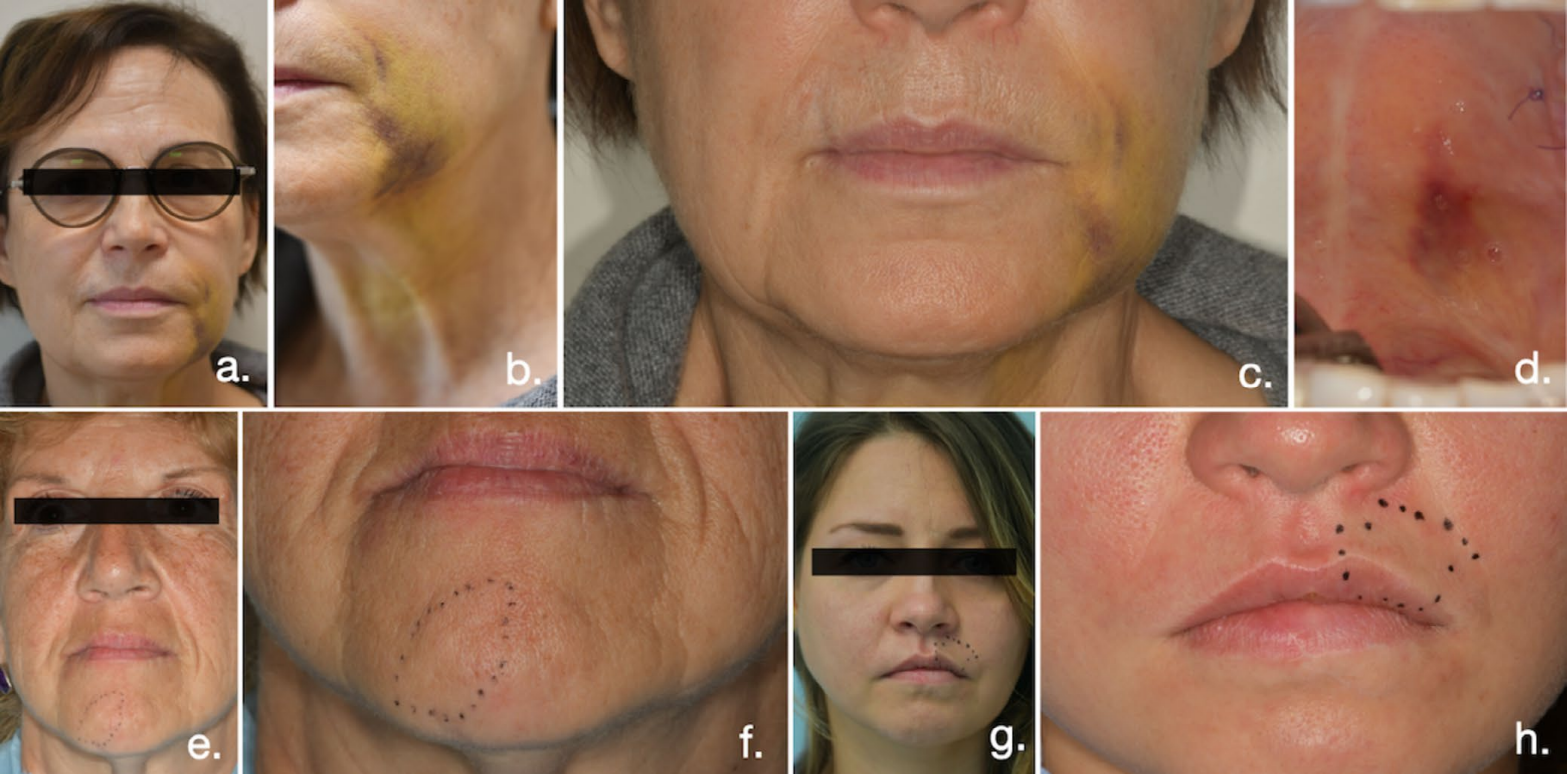
(a) Mean levels of post‐operative pain, recorded on a 100‐mm VAS scale ranging from 0 (‘no pain’) to 100 (‘worst pain imaginable’), entered in a daily diary over the 14 days following surgery, in the two study groups. (b) Mean dosage of anti‐inflammatory drugs entered in a daily diary over the course of the 14 days following surgery in the two study groups.

**FIGURE 6:**
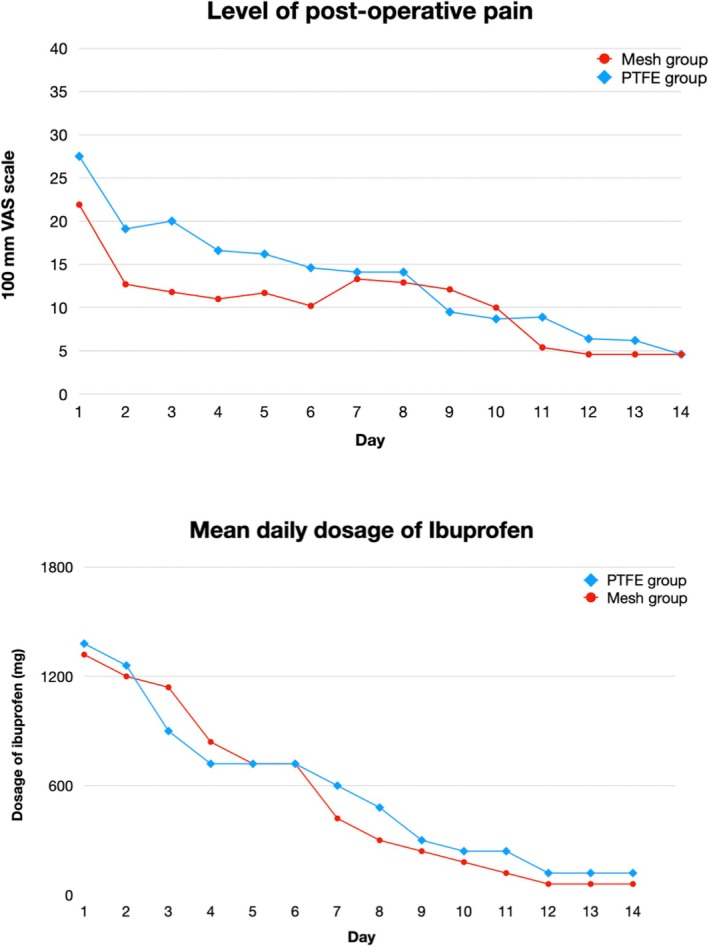
Clinical observation of post‐operative symptoms and complications representing patient's discomfort associated with the surgical procedure: (a–c) Post‐operative extraoral bruising area. (d) Post‐operative intraoral bruising area. (e–h) Identification of paresthesia areas marked on two different patients.

**FIGURE 7:**
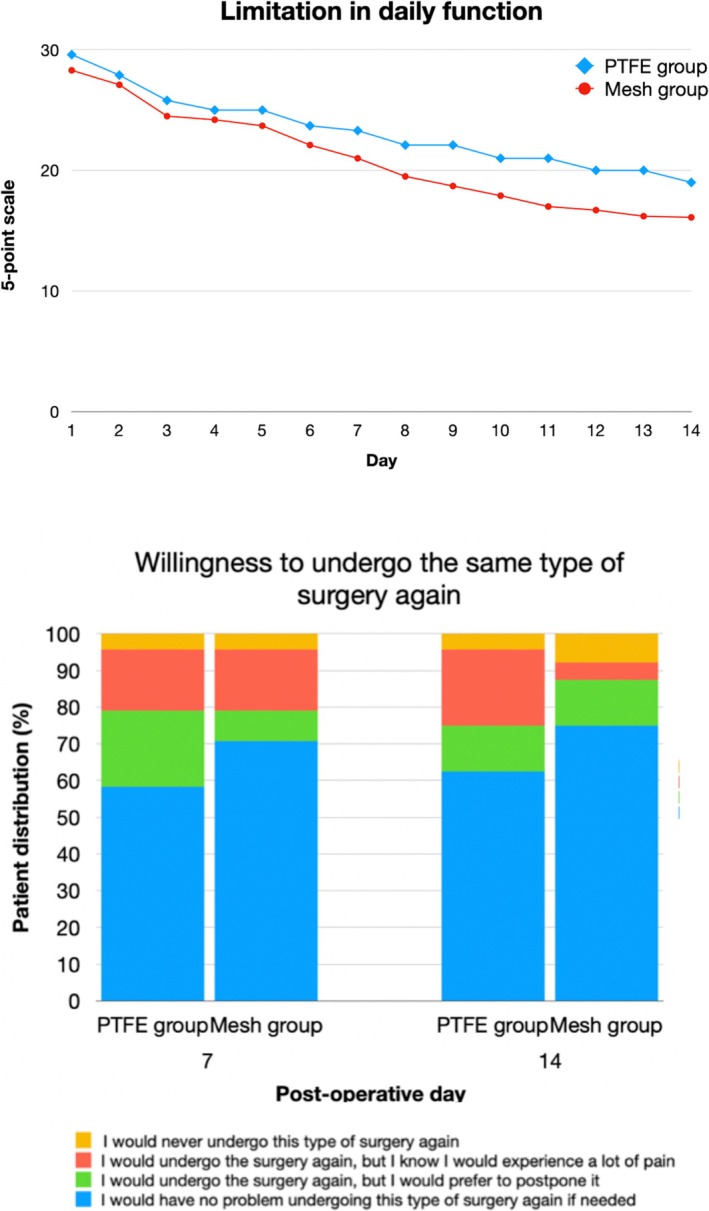
(a) Mean limitation in daily function recorded on a 5‐point scale ranging from 1 (‘not at all difficult’) to 5 (‘extremely difficult’) entered in a daily diary in the 14 days after surgery, in the two study groups. (b) Patient distribution according to willingness to undergo the same type of surgery if needed as recorded on a 4‐point scale at days 7 and 14 after surgery, from 1 (‘I would have no problem undergoing this type of surgery again if needed’) to 4 (‘I would never undergo this type of surgery again’).

1. Level of anxiety: The average of anxiety was 5.3 ± 2.8 in the PTFE group and 3.9 ± 3.3 in the Ti mesh group, pre‐operatively; these levels decreased to 1.9 ± 2.4 in PTFE group and 0.3 ± 0.7 in Ti mesh group, post‐operatively, with a significant reduction in both groups before and after surgery (*p* < 0.0001).
2. Treatment satisfaction: Similar mean OHIP values were found in both groups, with no statistically significant differences (*p* = 0.845); mean PoSSe values were comparable between the two groups, with no significant differences (*p* = 0.343). The mean PoSSe scores for discomfort were 35.7 ± 8.8 for the PTFE group and 33.2 ± 7.3 for the Ti mesh group (*p* = 0.343).
3. Levels of pain: This reduced from an average value of 2.7 ± 2.7 in the PTFE group and 2.2 ± 2.1 in the Ti mesh group on the day following surgery (*p* = 0.565) to 1.4 ± 2.0 and 1.3 ± 2.3 at 7 days (*p* = 0.263) and to 0.4 ± 0.7 and 0.4 ± 1.1 at 14 days after surgery (*p* = 0.328), respectively. There was no statistically significant differences between the two groups in any time intervals (Figure 5a).
4. The dosage of anti‐inflammatory drugs: This reduced from an average of 2.3 ± 1.2 in the PTFE group and 2.2 ± 1.0 in the Ti mesh group on the day following surgery (*p* = 0.599) to 1.04 ± 1.12 and 0.67 ± 0.76 at 7 days (*p* = 0.739) and to 0.2 ± 0.6 and 0.1 ± 0.3 at 14 days after surgery (*p* = 0.931), respectively. There was no statistically significant differences in any time intervals (Figure 5b).
5. Limitation in daily functions: It deceased from 2.9 ± 0.8 in the PTFE group and from 2.8 ± 0.8 in Ti mesh group on the following day (*p* = 0.608) to 2.33 ± 0.56 and 2.12 ± 0.99 at 7 days (*p* = 0.376), to 1.9 ± 0.7 in PTFE group and 1.6 ± 0.8 in Ti mesh group on day 14 after surgery(*p* = 0.085). The mean limitation in daily functions is shown in Figure 7a.
6. Patient distribution according to the incidence of post‐operative symptoms (swelling, nausea, bruises, bleeding, fever, lack of sensitivity, opening difficulties) is provided in Appendix S4 and Figure 6.
7. Willingness to undergo the same procedure again: At day 7, the means were 1.7 ± 0.9 and 1.5 ± 0.9 for the PTFE group and Ti mesh group, respectively (*p* = 0.529). At day 14, the means were 1.7 ± 0.9 and 1.4 ± 0.9, respectively (*p* = 0.301). Patient distribution according to willingness to undergo the same type of surgery if needed is shown in Figure 7b.

All data related to PROMs, including the level of pain, dosage of NSAIDs, limitation in function for each single day and univariate regression analysis, are shown in Appendix S4.

#### 3.1.5 Clinician‐Reported Outcomes Measures (ClinRO)

All ClinROs (level of anxiety, level of stress and level of satisfaction) are reported in Appendix S1. No differences were observed between the two study groups for any parameters. It is interesting to underline, for both groups, the medium/high level of anxiety of clinicians before surgery (6.4 of 10 and 6.3 of 10) and the high level of satisfaction after surgery (8.6 of 10 and 8.7 of 10).

#### 3.1.6 Univariate Linear Regression Analysis

All data related to the univariate linear regression analysis of the influencing factors are reported in Appendix S4. It is interesting to note that (i) the healing complications were related to high dosages of anti‐inflammatory drugs; (ii) the location (sextant/quadrant), defect size and defect depth (VDB) had a significant impact on intra‐operative time; (iii) the pain and anxiety levels of the patient influenced their willingness to undergo the same surgery again; and (iv) the dosage of anti‐inflammatory drugs, the level of pain and the severity of post‐operative symptoms were significantly interrelated.

## 4 Discussion

As demonstrated by many authors over the years and confirmed by a recent systematic review, GBR remains the most effective surgical procedure for VRA both in the mandible and maxilla (Cucchi et al. 2023) and the integration of technological innovation in dentistry has paved the way for personalised, digitally planned treatment approaches, leading to significant reductions in operative time, intra‐operative difficulties, post‐operative discomfort and pain (Urban et al. 2023; Alotaibi et al. 2023).

The inclusion of PROMs data enhances the understanding of the patient experience and offers valuable insights for both patients and clinicians when navigating through various procedures; they are progressively becoming pivotal endpoints in clinical trials (Vodicka et al. 2015). Although PROMs were reported in 20.2% of the prospective studies on horizontal and vertical bone augmentation, a recent systematic review found that PROMs were considered the primary outcome only in 2.2% of the cases (Shi et al. 2023).

The present study placed greater emphasis on collecting PROMs using validated tools to enhance the robustness and reliability of the findings. The results observed in both study groups showed consistent trends across both groups, with a decrease over time (from day 0 to day 14).

These findings also confirmed that anxiety related to the bone augmentation procedure remains a significant concern for patients, who reported a mean VAS score of 4.5 before the surgery. However, the sedation protocol used in this study confirms its efficacy in the reduction of anxiety to 1.5 on VAS after surgery. Post‐operative pain decreased in both groups from an average of 2.5 on the day following surgery, eventually nearly disappearing 14 days after surgery, and a complete resolution of post‐operative symptoms in 90% of patients after 2 weeks.

In this study, the OHIP‐14 questionnaire was administered to patients pre‐operatively, obtaining a mean value of approximately 12 points. On a scale ranging from 0 to 56, this relatively low score indicates a positive perception of the quality of life even prior to the bone augmentation treatment, considering that scores below 25 are considered ‘good’ (Chiapasco et al. 2018). Therefore, most of the patients would willingly undergo the same procedure again. More specifically, about 66% of patients would go through the surgery without any problems. These findings are consistent with the broader literature on patient‐reported outcomes in bone augmentation (Marconcini et al. 2019; Chiapasco et al. 2018; Mertens et al. 2013).

Regarding pre‐operative times, augmentation planning was executed similarly for all patients, and the average time required for virtual planning was similar in both groups. As to intra‐operative times, comparable total operative times emerged between the two groups, because the shaping and modelling of PTFE mesh were performed during a pre‐operative phase as previously described (Cucchi et al. 2022). Probably, without the aid of the pre‐modelled template (mesh replica) and the 3D stereolithographic model, this phase would have required more time, thus lengthening the surgical procedure by 10–20 min. This could increase the risk of infection due to the device.

Cost analysis confirms that GBR is an expensive technique. In Italy, the average cost is about €1200 with PTFE meshes and €1300 with customised Ti meshes. Digital planning for PTFE meshes simplifies modelling, saves time and lowers infection risk, but for experienced operators, skipping this step can save about €220. Comparing customised Ti meshes to conventional PTFE membranes shows a saving of about €400 (€800 vs. €1300). Further cost reductions are possible using reinforced PTFE membranes without perforations, thereby avoiding the need for a resorbable membrane. Studies directly comparing PTFE meshes and PTFE membranes are recommended.

It is crucial to acknowledge the limitations of this study. The sample size was based on a non‐inferiority design, and it is recommended to conduct larger scale investigations to further validate these findings. Another limitation could be the choice to use the novel PTFE meshes with a digital approach instead of conventional reinforced PTFE membranes as the control group. Then, the lack of a control group with conventional reinforced PTFE membranes is a limitation and does not allow one to draw conclusions on how digital technologies can improve the experience for the patient.

The following are the strengths of the study: The topic of the research, that is, VRA using two different digital approaches using novel barrier devices; randomised controlled design and strict analytical methodology; blinding of all people involved in the study (except for the second surgeon); the same operator for all surgeries; inclusion of both maxillary and mandibular sites; severity of the treated sites (mean VBG > 5.5 mm); novelty of the investigated parameters (PROMs, ClinROs, time, costs) in VRA procedures.

Therefore, VRA is seen to be a time‐ and cost‐intensive procedure, so biomaterial and operative times should be carefully evaluated and discussed with the patients. These findings have economic implications (times and costs) and highlight the importance of considering PROMs in addition to technical aspects when choosing between different approaches in GBR procedures. Further research in this domain will be essential for enhancing the overall quality of care in implant dentistry.

## 5 Conclusions

In conclusion, the comparative analysis of customised CAD/CAM Ti meshes and customised PTFE meshes, although requiring more pre‐operative planning and incurring higher costs, yielded similarly low complication rates and favourable PROMs, with no significant differences between the two study groups. These findings confirm the effectiveness and minimal invasiveness of the VRA procedure in the treatment of severe vertical defects.

## Author Contributions

**Alessandro Cucchi:** conceptualisation (lead), investigation (lead). **Sofia Bettini:** investigation (equal), methodology (equal), resources (supporting). **Lucia Tedeschi:** data curation (lead), writing – original draft (lead). **Debora Franceschi:** data curation (equal), writing – original draft (equal). **Istvan Urban:** methodology (supporting), supervision (supporting). **Antonino Fiorino:** formal analysis (lead), methodology (lead), software (lead), visualisation (lead). **Giuseppe Corinaldesi:** project administration (lead), supervision (lead).

## Conflicts of Interest

The authors declare no conflicts of interest.

## Supporting information


**Appendix S1.** Clinician‐reported outcomes measures (ClinRO).

**Appendix S2.** Statistical procedure.

**Appendix S3.** Supporting Information.

**Appendix S4.** Supporting Information.

## Data Availability

The data that support the findings of this study are available from the corresponding author upon reasonable request.
