# Supplementary material for: Operative Times, Costs and Patient‐Related Outcome Measures in Vertical Ridge Augmentation With Customised Reinforced PTFE Mesh Versus CAD/CAM Titanium Mesh: Secondary Analysis of a Randomised Clinical Trial
Source: J Clin Periodontol. 2025 May 26;52(7):971–82. doi: 10.1111/jcpe.14185 (PMC12176461; doi:10.1111/jcpe.14185)
Supplement: Supplementary file 1 — Appendix S1. Clinician‐reported outcomes measures (ClinRO). [file JCPE-52-971-s002.docx]

**APPENDIX S1**

*Clinician-reported outcomes measures (ClinRO)*

The clinician-reported outcomes measures (ClinRO) were assessed as follows:

1. level of anxiety, as recorded on a 100-mm visual analogue scale (VAS), reported 1, 2, 3 days before surgery, immediately before surgery and 14 days after surgery; (Hamilton, 1959)
2. Level of stress, measured with cardiovascular parameters (heart rate and blood pressure) every 20 minutes, during surgery, using a blood pressure monitor; (Rieger et al., 2014)
3. Level of satisfaction, as recorded on a 100-mm visual analogue scale (VAS), reported immediately after surgery, 14 and 30 days after surgery. (Meric, 1994).

The clinician-related outcome measures (anxiety, stress, satisfaction) were evaluated and analyzed using the same statistical analysis used for PROMs. The values measured in the 2 study groups were reported in tables A1.1-A1.4

In the PTFE group, the reported anxiety levels were 6.4±1.4 immediately before the operation and 5.1±2.6 at the 14-day follow-up; in the Ti-mesh group, the reported anxiety levels were 6.3±1.6 one day before surgery and 5.0±2.1 at the 14-day follow-up. No significant differences were observed between the 2 groups at any time interval. Mean anxiety levels of the operator are reported in Fig A1.1.

In the PTFE group, satisfaction levels were 8.6±1.2 at the end of surgery and 8.5±1.5 at 30 days; in the Ti-mesh group, satisfaction levels were 8.7±0.9 at the end of surgery and 8.8±1.5 at 30 days.

Similarly, no significant differences were observed between the 2 groups at any time interval. Mean satisfaction levels of the operator are reported in Fig. A1.2.

As for blood pressure, the average diastolic pressures were similar in the two groups (P=0.441), as well as the systolic pressures (P=0.407), without statistically significant differences. Similarly, the average heart rate showed no statistically significant differences in the two study groups (P=0.2198). Mean levels of the operator’s blood pressure and heart rate are listed in Fig A1.3 and Fig A1.4.

The clinician-reported outcomes showed that the surgeon experienced similar levels of anxiety and satisfaction throughout the study. While anxiety levels increased in the days leading up to surgery, they did not significantly differ between the two groups. This suggests that the introduction of the customization of PTFE mesh makes it possible to have similar stress values compared to customized Ti-meshes. The 3D analysis of the bone defects and the virtual planning of bone augmentation can help to reduce anxiety and stress related to VRA in both more- and less-experienced clinicians.

It is well recognized that VRA procedures are challenging and operator-dependent. As reported by Altoibi et al. (2023), there is a direct correlation between operator experience and the rate of complications in VRA techniques: high operator experience (>200 procedures) was directly linked to lower incidence of complications. In the present study, the first surgeon was moderately experienced in the treatment of severe vertical defects (about 200 procedures) and the level of anxiety was about 6 on VAS immediately before the surgery, confirming that VRA is a psychologically demanding procedure. Before the surgery, no differences were observed between the two groups, substantially due to the blindness of the surgeon; similarly, there were no differences in anxiety levels immediately and 14 days after surgery. Then, the satisfaction level measured post-operatively indicated the awareness to have concluded the surgery without mistakes or errors, complications, delays, or other problems, such as the difficulty in stabilizing the mesh or achieving a hermetic closure.

**Table A1.1.**

Clinician-reported outcome measures evaluated in the two study groups and overall.

| Variable | Control | | Test | | Overall | | p-value | Estimated  Mean (95% CI) |
| --- | --- | --- | --- | --- | --- | --- | --- | --- |
|  | **Mean ± SD (Median) 95% CI** | | **Mean ± SD (Median) 95% CI** | | **Mean ± SD (Median) 95% CI** | |  |  |
| Anxiety 3 Days Before Surgery | 4.54 ± 2.15 (5) | 3.64; 5.45 | 4.79 ± 2.17 | 3.88; 5.71 | 4.67 ± 2.14 (5) | 4.05; 5.29 | 0.7532  MW | 0.25 (-1.00, 1.50) |
| Anxiety 2 Days Before Surgery | 5.25 ± 1.87 | 4.46; 6.04 | 5.65 ± 1.34 | 5.08; 6.21 | 5.45 ± 1.62 | 4.98; 5.92 | 0.4037  t-test | 0.40 (-0.55, 1.34) |
| Anxiety 1 Day Before Surgery | 6.08 ± 1.56 | 5.43; 6.74 | 6.27 ± 1.61 | 5.59; 6.95 | 6.18 ± 1.57 | 5.72; 6.63 | 0.6836  t-test | 0.19 (-0.73, 1.11) |
| Anxiety Immediately Before Surgery | 6.46 ± 1.38 | 5.87; 7.04 | 6.67 ± 1.40 | 6.07; 7.26 | 6.56 ± 1.38 | 6.16; 6.96 | 0.6069  t-test | 0.21 (-0.60, 1.02) |
| Anxiety 14 Days After Surgery | 5.17 ± 2.65 | 4.05; 6.29 | 5.00 ± 2.09 | 4.12; 5.88 | 5.08 ± 2.36 | 4.40; 5.77 | 0.8097  t-test | -0.17 (-1.55, 1.22) |
| Satisfaction Immediately After Surgery | 8.65 ± 1.20 | 8.14; 9.15 | 8.73 ± 0.93 | 8.34; 9.12 | 8.69 ± 1.06 | 8.37; 9.00 | 0.7896  t-test | 0.08 (-0.54, 0.71) |
| Satisfaction 14 Days After Surgery | 7.92 ± 1.44 | 7.31; 8.53 | 8.10 ± 1.25 | 7.58; 8.63 | 8.01 ± 1.34 | 7.62; 8.40 | 0.4048  t-test | 0.10 (-0.84, 1.04) |
| Satisfaction 30 Days After Surgery | 8.58 ± 1.59 (9) | 7.91; 9.25 | 8.79 ± 1.50 (9) | 8.16; 9.43 | 8.69 ± 1.53 (9) | 8.24; 9.13 | 0.5617  MW | 0.21 (-0.69, 1.11) |

For each treatment group, the table shows the clinician anxiety and satisfaction (10 point VAS respectively) mean values at different time point. For each variable, the mean, standard deviation (SD), median (only for variables with non-normal distribution), 95% confidence interval (95%CI) were reported. MW: Mann-Whitney test

**Table A1.2.**

Levels of the operator’s minimum systolic blood pressure measured every 20 minutes during surgery evaluated in the two study groups and overall.

| Variable | Control  (Mean ± SD, Median) 95% CI | | Test  (Mean ± SD, Median) 95% CI | | Overall  (Mean ± SD, Median) 95% CI | | p-value | Estimated Difference  (Mean, 95% CI) |
| --- | --- | --- | --- | --- | --- | --- | --- | --- |
| PAMini1 | 79.67 ± 6.54 | 76.91; 82.42 | 76.42 ± 11.99 | 71.36; 81.48 | 78.04 ± 9.69 | 75.23; 80.86 | 0.2496  t-test | -3.25 (-8.86; 2.36) |
| PAMini2 | 80.92 ± 10.22 (82.5) | 76.60; 85.23 | 76.92 ± 10.71 (78) | 72.39; 81.44 | 78.92 ± 10.55 (79) | 75.85; 81.98 | 0.1064 MW | -4.00 (-10.08; 2.08) |
| PAMini3 | 80.33 ± 8.32 | 76.82; 83.85 | 80.54 ± 10.36 | 76.17; 84.92 | 80.44 ± 9.30 | 77.74; 83.14 | 0.9391  t-test | 0.21 (-5.25; 5.67) |
| PAMini4 | 77.71 ± 8.35 | 74.18; 81.24 | 79.63 ± 10.01 | 75.40; 83.85 | 78.67 ± 9.17 | 76.00; 81.33 | 0.4751  t-test | 1.92 (-3.44; 7.27) |
| PAMini5 | 76.17 ± 9.32 (76.5) | 72.23; 80.10 | 76.42 ± 12.45 (78) | 71.16; 81.67 | 76.29 ± 10.88 (77.5) | 73.13; 79.45 | 0.6860  MW | 0.25 (-6.14; 6.64) |
| PAMini6 | 77.83 ± 14.11 (74) | 70.81; 84.85 | 78.36 ± 8.35 (78) | 74.66; 82.06 | 78.13 ± 11.15 (78) | 74.56; 81.69 | 0.3480  MW | 0.53 (-6.74; 7.80) |
| PAMini7 | 76.54 ± 6.89 | 72.38; 80.70 | 77.29 ± 8.73 | 72.81; 81.78 | 76.97 ± 7.86 | 74.03; 79.90 | 0.7994  t-test | 0.76 (-5.28; 6.79) |
| PAMini8 | 79.58 ± 23.34 (76.5) | 64.75; 94.41 | 76.83 ± 8.13 (78.5) | 71.67; 82.00 | 78.21 ± 17.15 (77) | 70.97; 85.45 | 0.5793  MW | -2.75 (-17.55; 12.05) |
| PAMini9 | 71.20 ± 4.55 | 65.55; 76.85 | 78.83 ± 5.81 | 72.74; 84.93 | 75.36 ± 6.41 | 71.06; 79.67 | 0.0410*  t-test | 7.63 (0.39; 14.88) |
| PAMini10 | 71.00 ± 2.25 | 68.42, 73.58 | 75.33 ± 2.08 | 70.16; 80.50 | 74.25 ± 3.25 | 69.87; 78.63 | 0.0752  t-test | 4.33 (2.33; 7.02) |

For each treatment group, the table shows the operator’s minimum systolic blood pressure mean values at different time point. For each variable, the mean, standard deviation (SD), median (only for variables with non-normal distribution), 95% confidence interval (95%CI) were reported. *Statistically significant difference. MW: Mann-Whitney test.

**Tab A1.3.**

Levels of the operator’s maximum systolic blood pressure measured every 20 minutes during surgery evaluated in the two study groups and overall.

| Variable | Control  (Mean ± SD, Median) 95% CI | | Test  (Mean ± SD, Median) 95% CI | | Overall  (Mean ± SD, Median) 95% CI | | p-value | Estimated Difference  (Mean, 95% CI) |
| --- | --- | --- | --- | --- | --- | --- | --- | --- |
| PAMaxi1 | 127.29 ± 9.66 (127.5) | 122.92; 131.99 | 126.83 ± 14.96 (124.5) | 120.52; 133.15 | 127.06 ± 12.46 (125.5) | 123.45; 130.68 | 0.4155  MW | -0.46 (-7.77; 6.86) |
| PAMaxi2 | 127.46 ± 10.74 (127.5) | 122.92; 131.99 | 119.21 ± 23.42 (124.5) | 109.32; 129.10 | 123.33 ± 18.50 (125.5) | 117.96; 128.70 | 0.1828 MW | -8.25 (-18.84; 2.34) |
| PAMaxi3 | 131.38 ± 7.80 | 128.08; 134.67 | 129.04 ± 9.23 | 125.14; 132.94 | 130.21 ± 8.54 | 127.73; 132.69 | 0.3493  t-test | -2.33 (-7.30; 2.63) |
| PAMaxi4 | 132.54 ± 11.62 | 127.63; 137.45 | 126.58 ± 10.36 | 122.21; 130.96 | 129.56 ± 11.30 | 126.28; 132.84 | 0.0672  t-test | -5.96 (-12.36; 0.44) |
| PAMaxi5 | 127.00 ± 11.39 (124.5) | 122.19; 131.81 | 124.92 ± 16.27 (127) | 118.05; 131.79 | 125.96 ± 13.93 (125.5) | 121.91; 130.00 | 0.3645  MW | -2.08 (-10.24; 6.08) |
| PAMaxi6 | 123.09 ± 10.88 | 118.27; 127.91 | 127.94 ± 13.51 (126) | 121.22; 134.67 | 125.28 ± 12.22 (125) | 121.37; 129.18 | 0.5668  MW | -4.85 (-12.66; 2.95) |
| PAMaxi7 | 123.85 ± 9.63 | 118.02; 129.67 | 127.06 ± 5.93 | 124.01; 130.11 | 125.67 ± 7.77 | 122.76; 128.57 | 0.1347  t-test | 3.21 (-2.63; 9.05) |
| PAMaxi8 | 127.25 ± 17.39 (128.5) | 116.20; 138.30 | 125.33 ± 8.19 (124) | 120.13; 130.54 | 126.29 ± 13.33 (125) | 120.66; 131.92 | 0.5604  MW | -1.92 (-13.43; 9.59) |
| PAMaxi9 | 128.50 ± 11.98 | 115.93; 141.07 | 120.20 ± 9.60 | 108.28; 132.12 | 124.73 ± 11.29 (125) | 117.14; 132.31 | 0.2436  t-test | 8.30 (-6.75; 23.35) |
| PAMaxi10 | 125.00 ± 11.27 | 97.01; 152.99 | 123.00 ± 13.5 | 119.0; 130.0 | 124.75 ± 12.43 | 122.284; 127.216 | 0.598  t-test | 0.5 (-4.44; 5.44) |

For each treatment group, the table shows the operator’s maximum systolic blood pressure mean values at different time point. For each variable, the mean, standard deviation (SD), median (only for variables with non-normal distribution), 95% confidence interval (95%CI) were reported.

MW: Mann-Whitney test.

**Table A1.4.**

Levels of the operator’s heart rate (measured every 20 minutes during surgery) evaluated in the two study groups and overall.

| Variable | Control  (Mean ± SD, Median) 95% CI | | Test  (Mean ± SD, Median) 95% CI | | Overall  (Mean ± SD Median) 95% CI | | p-value | Estimated Difference  (Mean, 95% CI) |
| --- | --- | --- | --- | --- | --- | --- | --- | --- |
| HRi1 | 82.79 ± 8.21 | 79.33; 86.26 | 79.38 ± 9.55 | 75.34; 83.41 | 81.08 ± 8.98 | 78.48; 83.69 | 0.1904 t-test | -3.42 (-8.59; 1.76) |
|  |  |  |  |  |  |  |  |  |
| HRi2 | 85.17 ± 8.79 | 81.46; 88.88 | 82.46 ± 11.05 (79) | 77.79; 87.12 | 83.81 ± 9.97 | 80.92; 86.71 | 0.2116  MW | -2.71 (-8.51; 3.09) |
|  |  |  |  |  |  |  |  |  |
| HRi3 | 85.54 ± 9.10 | 81.70; 89.38 | 80.54 ± 10.81 | 75.98; 85.10 | 83.04 ± 10.20 | 80.08; 86.00 | 0.0896  t-test | -5.0 (-10.8; 0.8) |
|  |  |  |  |  |  |  |  |  |
| HRi4 | 84.5 ± 8.64 | 80.85; 88.15 | 82.0 ± 8.32 | 78.48; 85.52 | 83.25 ± 8.49 | 80.79; 85.71 | 0.3128  t-test | -2.5 (-7.43; 2.43) |
|  |  |  |  |  |  |  |  |  |
| HRi5 | 84.42 ± 9.64 | 80.35; 88.49 | 81.08 ± 7.19 | 78.05; 84.12 | 82.75 ± 8.58 | 80.26; 85.24 | 0.1811  t-test | -3.33 (-8.27; 1.61) |
|  |  |  |  |  |  |  |  |  |
| HRi6 | 83.06 ± 8.00 | 79.08; 87.04 | 80.77 ± 8.82 | 76.86; 84.68 | 81.8 ± 8.43 | 79.10; 84.50 | 0.4013  t-test | -2.28 (-7.73; 3.16) |
|  |  |  |  |  |  |  |  |  |
| HRi7 | 80.08 ± 7.90 | 75.30; 84.85 | 81.59 ± 7.18 | 77.40; 85.77 | 80.93 ± 7.93 | 77.97; 83.90 | 0.6138  t-test | 1.51 (-4.56; 7.58) |
|  |  |  |  |  |  |  |  |  |
| HRi8 | 79.0 ± 6.12 | 75.11; 82.89 | 83.25 ± 7.56 | 78.45; 88.05 | 81.13 ± 7.07 | 78.14; 84.11 | 0.1443  t-test | 4.25 (-1.57; 10.07) |
|  |  |  |  |  |  |  |  |  |
| HRi9 | 76.4 ± 7.20 (78) | 67.46; 85.34 | 81.17 ± 8.13 | 72.63; 89.70 | 79.0 ± 7.75 | 73.80; 84.20 | 0.2684  MW | 4.77 (-5.82; 15.36) |
|  |  |  |  |  |  |  |  |  |
| HRi10 | 91.0 ± 10.44 (91) | 53.87; 111.46 | 82.67 ± 11.59 (81) | 53.87; 111.46 | 84.75 ± 10.34 (86) | 68.30; 101.20 | 0.7482  MW | -8.33 (- 15.78 ; 0.88) |

For each treatment group, the table shows the operator’s heart rate values at different time point. For each variable, the mean, standard deviation (SD), median (only for variables with non-normal distribution), 95% confidence interval (95%CI) were reported.

MW: Mann-Whitney test.


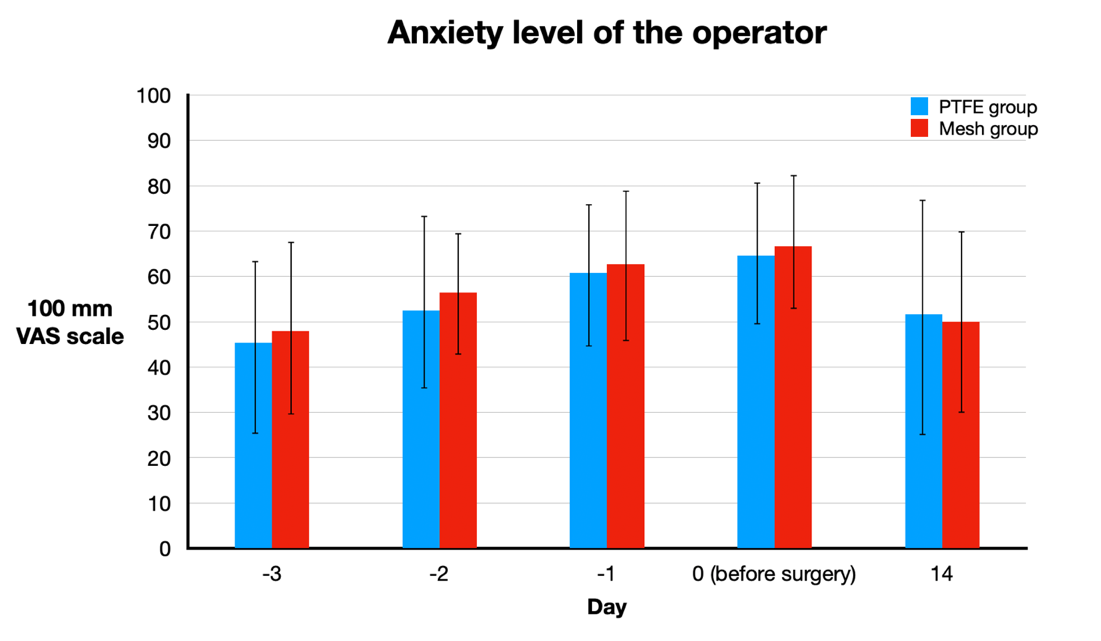


**Figure A1.1.**

Mean operator's anxiety levels recorded on a 100-mm VAS scale, from “1, not at all stressed” to “100, extremely stressed” reported 1, 2, 3 days before surgery, immediately before surgery and 14 days after surgery, in the two study groups.


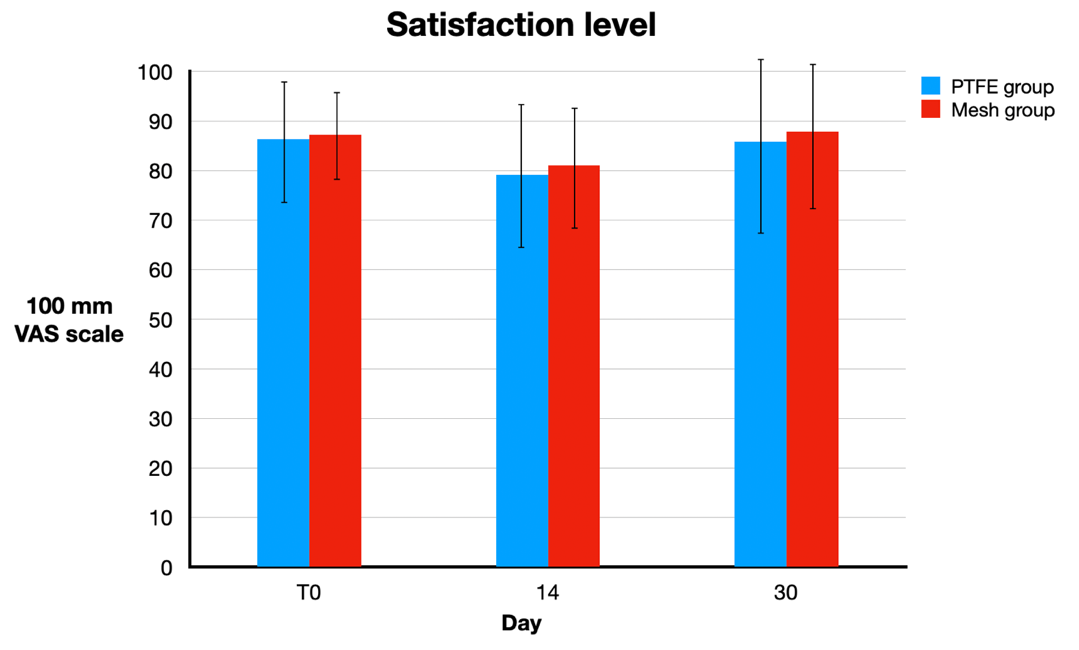


**Figure A1.2.**

Mean operator satisfaction levels recorded on a 100-mm VAS scale from “1, not at all satisfied” to “100, extremely satisfied”, reported immediately after surgery, 14 days and 30 days after surgery, in the two study groups.

**Figure A1.3.**

Mean levels of the operator’s maximum and minimum systolic blood pressure measured every 20 minutes during surgery, using a blood pressure monitor, in the two study groups.


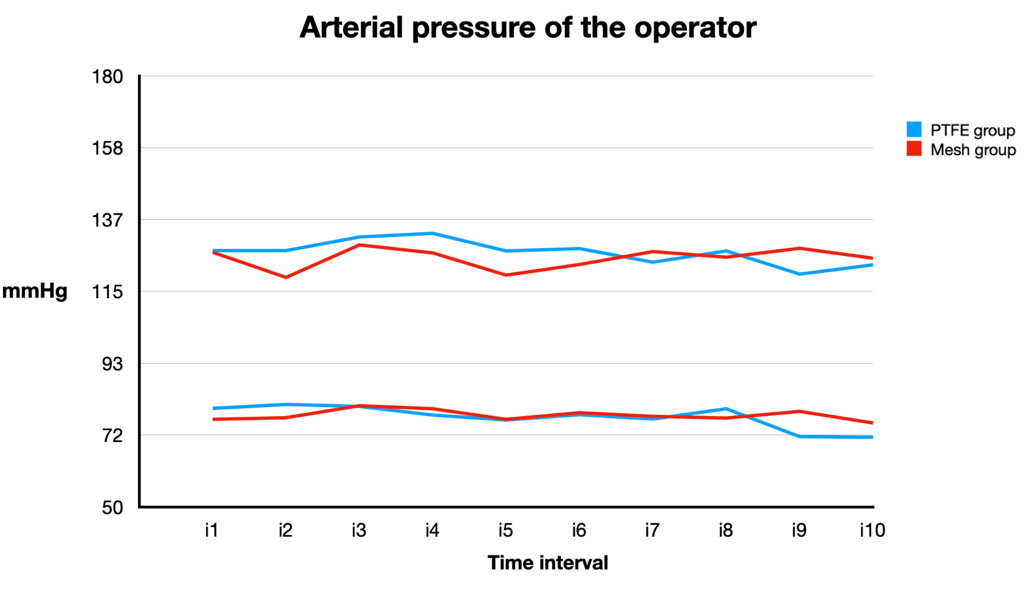


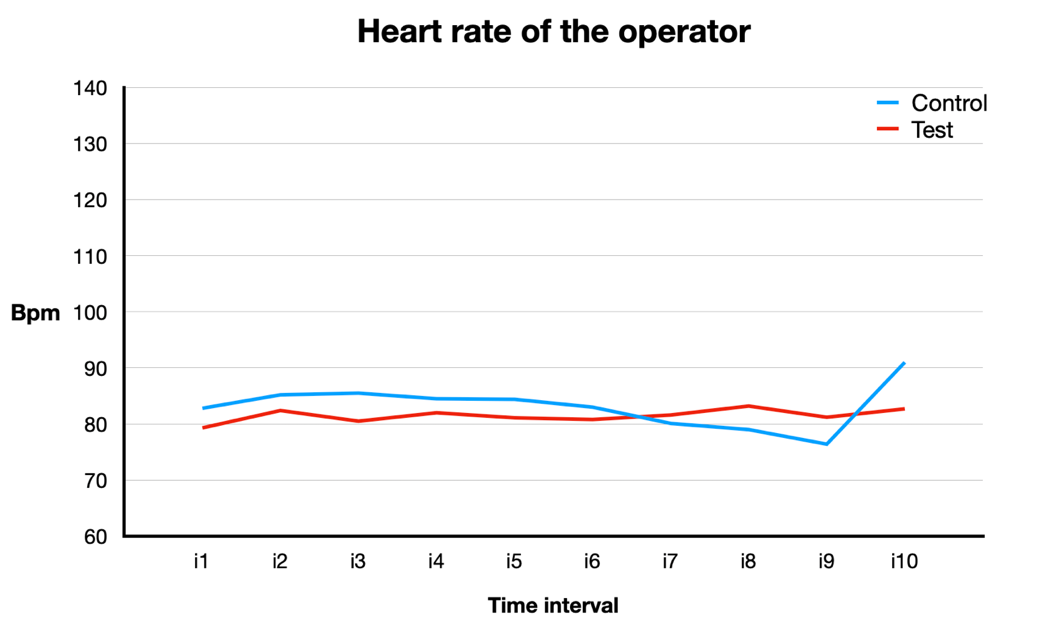


**Figure A1.4.**

Mean levels of the operator’s heart rate measured every 20 minutes, during surgery, using a blood pressure monitor, in the two study groups.
