# Supplementary material for: Operative Times, Costs and Patient‐Related Outcome Measures in Vertical Ridge Augmentation With Customised Reinforced PTFE Mesh Versus CAD/CAM Titanium Mesh: Secondary Analysis of a Randomised Clinical Trial
Source: J Clin Periodontol. 2025 May 26;52(7):971–82. doi: 10.1111/jcpe.14185 (PMC12176461; doi:10.1111/jcpe.14185)
Supplement: Supplementary file 2 — Appendix S2. Statistical procedure. [file JCPE-52-971-s003.docx]

**APPENDIX S2**

*Statistical procedure*

Excel data collection form and a data management system were used (Microsoft Excel 2011; Windows, ver. 14.0.0; Microsoft Corp.). All data were entered by a single blinded operator. Prior to entry, all data were evaluated in terms of accuracy and completeness. The mean, median (only if the distribution was non-normal), standard deviation (SD) and 95% confidence interval (95% CI) were reported for each continuous variable. This statistical analysis, calculated at the patient level, was based on the hypothesis (H1) that Ti-mesh (test group) would not be inferior to reinforced PTFE mesh (control group) in the incidence of healing complications (primary outcome).

The non-inferiority test was performed for Healing and Surgical complication only (one-sided 95% confidence interval approach), specifying the greatest difference that was clinically acceptable for each outcome (delta = D). Regarding the non-inferiority limit, in accordance with CONSORT guidelines, the authors identified the upper limit of the CI for the Test Group and the lower limit of the CI for the Control Group. These values served as the basis for calculating the maximum allowable difference. The resulting margin was graphically positioned to the right, indicating that the Ti-Mesh Group is not considered inferior to the PTFE Group if the mean difference in quantity does not exceed 10% of clinical significance. In addition, superiority analyses were carried out to evaluate significant differences in terms of intervention group and all other variables. The assumption of normality was assessed for the intervention group and overall, on all continuous numerical variables through Skewness/Kurtosis tests (normal distribution if p-value > 0.05). The comparison of means to evaluate statistically significant differences was performed by the t-test, the Wilcoxon matched-pairs signed-ranks test and the Wilcoxon rank-sum test (Mann-Whitney test) when necessary.

For qualitative data frequencies, proportions and 95% confidence intervals for proportions were calculated. In bivariate analysis, proportions were compared using two tests. The chi-square tests (i.e., χ2-test) were performed when no >20% of the cells of the contingency tables had frequencies of 5 or less and that no cells had expected frequencies <1 (Cochran, 1954). If any of the observed values was <5, then a Fisher's exact test was performed. A test of normality was carried out with the Skewness/Kurtosis tests (normal distribution if p > .05). Categorical variables were also evaluated numerically, classes from 0 to 1 (healing, surgical complications and all post-operative symptoms) and from 1 to 4 (willingness to undergo the same type of surgery again). The threshold value for statistical significance was determined setting a p-value of 0.05 (5%).
Univariate linear regression analyses were conducted to assess the relationships between healing, surgical complications, total intraoperative time, POSSE, NSAIDs, PAIN, and willingness to undergo the same type of surgery with other factors. The results are presented with R-squared values to indicate the proportion of variance explained, 95% confidence intervals (CI) to reflect the precision of the estimates, and p-values to determine the statistical significance of the findings. Analyses were performed using STATA, with a significance threshold set at p < 0.05. Data analysis was performed with Stata/IC software (StataCorp LLC).

*Non-inferiority Study Design*

Since there were no studies comparing customized Ti-meshes and Ti-PTFE meshes, the non-inferiority design has been considered the most ethical approach for patients. The main reasons why the present authors have chosen a non-inferiority design for this RCT are:

- Ethical Considerations: In cases where the standard treatment is effective (in this case, PTFE; there are a lot of randomized and non-randomized studies about PTFE, but there are very few studies about CAD/CAM titanium meshes) and withholding it could harm participants, a non-inferiority trial can ensure the new treatment is not worse than the standard by a clinically acceptable margin.
- Practicality in Established Treatments: When there is already an established effective treatment (in this case, PTFE), proving a new treatment is superior can be very challenging and might require a large sample size. Instead, demonstrating that the new treatment is not significantly worse (non-inferior) can be more feasible. Up to now, there are no previous studies with similar design, so a pilot study should be carried out. However, the authors decided to carry out a non-inferiority RCT in order to have a larger sample of patients and more data for analysis.
- Innovations in Treatment: Non-inferiority trials are particularly useful when the new treatment offers other benefits, such as fewer side effects, lower cost, easier administration/application, or improved quality of life. In this case, CAD/CAM titanium meshes could offer fewer side effects (pain, discomfort, symptoms) or easier application (times and handling); if it is not inferior, the treatment can still be preferable respect to the control.
- Regulatory Acceptance: The ethical committee (Institutional Review Board for Human Studies of the University of Szeged) has accepted more favorable the non-inferiority study, as evidence for approval when a new treatment needs to demonstrate that it is at least as good as an existing treatment. This is common in areas where treatments are well-established, and the focus is on maintaining efficacy while improving other aspects of care.
- Clinical Relevance: In many situations, clinicians and patients may be more interested in knowing that a new treatment is not worse than the standard treatment by a meaningful amount rather than being slightly better. This approach aligns with the real-world clinical decision-making process.
- Economic and Logistical Advantages: Non-inferiority trials can be more cost-effective and require fewer resources compared to superiority trials. This is because proving non-inferiority often requires a smaller sample size than proving superiority, especially when the difference expected is small.

In summary, a non-inferiority analysis can be more appropriate in settings where there is an established effective treatment (Ti-PTFE, as previously published), and the goal is to show that the new treatment is not significantly worse, especially when it could offer other benefits (lower complication, lower economic cost, shorter operative time, more favorable PROMs,…). This approach can align better with ethical standards, regulatory requirements, clinical relevance, and practical considerations in clinical research.

*Per-Protocol Analysis (PP)*

A PP analysis was chosen to describe the collected data and to explore the results, so only patients that followed strictly the study protocol were included in the statistical analysis.

In details, the reasons of choice PP analysis compared to Intent-to-treat (ITT) analysis are the following. First of all, PP analysis can provide a more accurate estimation of the treatment effect as it includes only those participants who adhered to the protocol, (i) ensuring that the observed effects are due to the treatment itself rather than external factors and (ii) reducing the risk of bias due to non-compliance or protocol deviations. Then, PP analysis may offer a realistic assessment of how effective a treatment is under optimal conditions, reflecting the maximum potential benefit of the intervention. It focuses on participants who actually used the intervention as intended, which can provide valuable information for clinicians about the effectiveness of the treatment in a setting where adherence is expected.

Nevertheless, the ITT approach remains the traditionally preferred strategy for analyzing and interpreting randomized trials. In ITT analysis, the outcomes of groups formed through randomization are compared without regard to whether participants actually received the assigned treatment or adhered to other protocol requirements. This approach helps preserve the integrity of the original randomization, reducing the potential for bias that can arise from excluding participants. By including all randomized participants in the analysis, regardless of their adherence to the treatment protocol, the ITT approach provides a more accurate reflection of real-world effectiveness and enhances the generalizability of the trial results.
