# Supplementary material for: Operative Times, Costs and Patient‐Related Outcome Measures in Vertical Ridge Augmentation With Customised Reinforced PTFE Mesh Versus CAD/CAM Titanium Mesh: Secondary Analysis of a Randomised Clinical Trial
Source: J Clin Periodontol. 2025 May 26;52(7):971–82. doi: 10.1111/jcpe.14185 (PMC12176461; doi:10.1111/jcpe.14185)
Supplement: Supplementary file 3 — Appendix S3. Supporting Information. [file JCPE-52-971-s001.docx]

**APPENDIX S3**

In the 50 treated patients, a total of 26 mandibular defects and 22 maxillary defects were treated. All treated defects were partial edentulous sites. Defect geometry was classified as “combined” horizontal-vertical in 24 cases and purely vertical in 24 cases. Of the treated defects, 3 were classified as small (1 tooth), 31 as medium (2 to 3 teeth), and 14 as large (more than 3 teeth). According to Kennedy’s classification, 3 patients were classified as class I, 29 as class II, 7 as class III, and 9 as class IV.

Therefore, 42 patients were non-smokers and 6 patients smoked fewer than 10 cigarettes per day; no patient smoked more than 10 cigarettes a day; 32 patients had no periodontal disease, while the 16 patients with periodontitis were treated with periodontal therapy before augmentation surgery.

Out of 48 patients that completed the augmentation surgery (T0), 5 patients experienced a healing complication and a partial or complete graft loss (T1).

For each patient, the vertical bone defect (VBD) was linearly measured in millimeters (mm) using dedicated software (ImageJ, NIH) on the cross-sectional images of pre-operative CBCT, focusing on the area of maximum defect in relation to the “ideal” line of regeneration; the distance from the maximum defect point to the nearby tooth was measured and noted as the reference point for the post-operative measurements. In post-operative CBCT, the corresponding cross-sectional image was then used to assess the vertical bone gain (VBG) in millimeters (mm). The ratio between VBG and VDB was used to calculate the linear regeneration rates (%).

At the surgery (T0), the VBD measurements were 6.08 ± 1.77 mm, ranging from 3.7 to 9.10 mm in the PTFE group, and 5.59 ± 1.68 mm, with a range from 3.10 to 9.40 mm in the Ti-mesh group (p=0.359).

At the time of re-entry surgery (T1), the VBG measurements were 5.79 ± 1.71 mm, ranging from 3.2 to 8.8 mm in the PTFE group, and 5.18 ± 1.61 mm, with a range from 3.1 to 8.0 mm in the Ti-mesh group (p=0.233). The linear regeneration rates (%) were 95.4 ± 7.6% and 92.6 ± 6.4% for the PTFE and Ti-mesh groups, respectively (p=0.134).

All the measurements are reported in table A3.1.

**Table A3.1**

Clinical characteristics of each patient in the PTFE group, including patient identification code, gender, age, ASA classification, periodontal health, smoking status, defect size, defect type (pure vertical V; combined horizontal-vertical HV) and Kennedy's classification.

| **N** | **Gender** | **Age** | **ASA** | **Periodontitis** | **Smoking** | **Defect size** | **Defect type** | **Kennedy class** |
| --- | --- | --- | --- | --- | --- | --- | --- | --- |
| 1 | F | 68 | 2 | + | - | Medium | V | II |
| 2 | M | 61 | 1 | - | - | Large | V | II |
| 4 | F | 64 | 1 | - | - | Large | V | II |
| 5 | M | 64 | 2 | - | - | Large | V | II |
| 6 | F | 36 | 1 | - | - | Small | HV | II |
| 7 | M | 33 | 2 | + | + | Medium | V | IV |
| 11 | F | 63 | 1 | - | + | Medium | V | II |
| 13 | M | 62 | 3 | + | - | Medium | HV | II |
| 15 | F | 50 | 2 | - | - | Medium | V | II |
| 17 | M | 39 | 1 | - | + | Medium | V | II |
| 19 | F | 59 | 1 | - | - | Medium | HV | II |
| 20 | F | 64 | 2 | - | - | Large | HV | II |
| 22 | F | 51 | 1 | - | + | Medium | V | III |
| 25 | F | 59 | 1 | - | - | Medium | HV | II |
| 26 | F | 60 | 1 | - | - | Medium | HV | II |
| 28 | F | 57 | 2 | - | - | Large | HV | II |
| 38 | F | 43 | 2 | - | + | Medium | V | III |
| 40 | F | 61 | 3 | + | - | Medium | HV | II |
| 41 | F | 75 | 2 | + | - | Medium | V | II |
| 43 | M | 32 | 1 | - | - | Small | V | III |
| 44 | M | 41 | 1 | + | - | Large | HV | IV |
| 46 | F | 41 | 1 | - | - | Medium | HV | IV |
| 47 | F | 79 | 1 | - | - | Large | V | II |
| 48 | M | 55 | 1 | + | - | Large | HV | II |
| 50 | M | 62 | 1 | + | - | Large | HV | II |

**Table A3.2**

Clinical characteristics of each patient in the Ti-mesh group, including patient identification code, gender, age, ASA classification, periodontal health, smoking status, defect size defect type (pure vertical V; combined horizontal-vertical HV) and Kennedy's classification.

| **N** | **Gender** | **Age** | **ASA** | **Periodontitis** | **Smoking** | **Defect size** | **Defect type** | **Kennedy class** |
| --- | --- | --- | --- | --- | --- | --- | --- | --- |
| 3 | F | 53 | 2 | - | - | Medium | HV | II |
| 8 | F | 62 | 1 | - | - | Medium | HV | II |
| 9 | F | 70 | 3 | - | - | Medium | V | II |
| 10 | F | 30 | 1 | - | - | Medium | V | IV |
| 12 | F | 49 | 2 | + | + | Medium | V | II |
| 14 | F | 36 | 1 | + | - | Medium | V | II |
| 16 | F | 54 | 2 | - | - | Medium | HV | II |
| 18 | F | 71 | 1 | + | - | Medium | HV | III |
| 21 | F | 30 | 1 | - | - | Large | V | IV |
| 23 | F | 57 | 2 | - | - | Large | HV | II |
| 24 | F | 36 | 1 | - | - | Medium | HV | IV |
| 27 | M | 64 | 1 | - | - | Medium | V | II |
| 29 | F | 59 | 1 | - | - | Small | V | III |
| 30 | M | 54 | 1 | - | - | Medium | V | II |
| 31 | F | 62 | 1 | + | - | Large | HV | II |
| 32 | F | 52 | 2 | + | - | Large | HV | II |
| 33 | F | 59 | 3 | - | - | Medium | HV | II |
| 34 | F | 60 | 1 | - | - | Medium | V | II |
| 35 | M | 69 | 1 | + | - | Medium | HV | IV |
| 36 | F | 67 | 1 | + | - | Medium | HV | II |
| 37 | F | 71 | 2 | - | - | Medium | V | II |
| 39 | M | 54 | 1 | - | - | Medium | V | II |
| 42 | F | 65 | 1 | - | - | Medium | HV | IV |
| 45 | M | 63 | 1 | - | - | Medium | HV | II |
| 49 | F | 74 | 2 | + | - | Large | V | II |

**Table A3.3**

Demographic and clinical characteristics of patients evaluated in the two study groups and in the total population, including age (mean), gender (n. and percentage), ASA classification (n. and percentage), periodontal health (n. and percentage), smoking status (n. and percentage), defect location (n. and percentage), defect size (n. and percentage), defect type (n. and percentage), Kennedy's classification (n. and percentage), Vertical Bone Gain (VBG) (mean), Regeneration Rate (RR) (percentage).

|  | PTFE Group | Ti-mesh Group | Total |
| --- | --- | --- | --- |
| Age |  |  |  |
|  | 55 | 57 | 56 |
| Gender |  |  |  |
| M | 9, 36% | 5. 20% | 14. 28% |
| F | 16. 64% | 20, 80% | 36. 72% |
| ASA |  |  |  |
| 1 | 14. 56% | 15. 60% | 29. 58% |
| 2 | 8. 32% | 7. 28% | 15. 30% |
| 3 | 2. 8% | 2. 8% | 4. 8% |
| Periodontitis |  |  |  |
| yes | 8. 32% | 8. 32% | 16. 32% |
| no | 17. 68% | 17. 68% | 34. 68% |
| Smoking |  |  |  |
| Less than 10 cig/die | 5. 20% | 1. 4% | 6. 12% |
| No smokers | 20, 80% | 24. 96% | 44. 88% |
| Defect location |  |  |  |
| Maxilla | 10, 41.7% | 12. 50% | 22. 45.8% |
| Mandible | 14. 58.3% | 12. 50% | 26. 54.2% |
| Defect Size |  |  |  |
| Small | 2. 8.3% | 1. 4.2% | 3. 6.2% |
| Medium | 13. 54.2% | 18. 75% | 31. 64.6% |
| Large | 9. 37.5% | 5. 20.8% | 14. 29.2% |
| Defect type |  |  |  |
| Purely vertical | 13. 54.2% | 11. 45.8% | 24. 50% |
| Combined | 11. 45.8% | 13. 54.2% | 24. 50% |
| Kennedy’s Class |  |  |  |
| I | 3. 12.5% | 2. 8.3% | 3. 6.2% |
| II | 12. 50% | 15. 62.5% | 29. 60.4% |
| III | 5. 20.8% | 2. 8.3% | 7. 14.6% |
| IV | 4. 16.7% | 5. 20.8% | 9. 18.7% |
| VBG (mm) |  |  |  |
|  | 5.80 mm | 5.18 mm | 5.48 mm |
| RR (%) |  |  |  |
|  | 99.5 % | 87.0 % | 93.09 % |

**Table. A3.4**

Volumes measurements evaluated in the two study groups and overall.

| Variable | Control | | Test | | Overall | | p-value | Estimated |
| --- | --- | --- | --- | --- | --- | --- | --- | --- |
|  | Mean ± SD (Median) | 95% CI | Mean ± SD (Median) | 95% CI | Mean ± SD (Median) | 95% CI |  | Mean (95% CI) |
| Linear RR (%) | 95.43 ± 5.75 (97.70) | 92.81; 98.05 | 92.59 ± 6.36 (93.58) | 89.77; 95.41 | 93.98 ± 6.17 (95.89) | 92.08; 95.88 | 0.0524  MW | -2.83 (CI: -6.57; 0.91) |
| VBG (mm) | 5.79 ± 1.71 | 5.01; 6.58 | 5.18 ± 1.61 | 4.47; 5.90 | 5.48 ± 1.67 | 4.97; 6.00 | 0.2334  t-test | -0.61 (CI: -1.6; 0.4109) |

For each treatment group, the table presents the mean values of Vertical Bone Gain (VBG) in millimeters and Regeneration Rate (RR) as a percentage. For each variable, the mean, standard deviation (SD), median (for variables with non-normal distribution only), and 95% confidence interval (95% CI) are reported. *Statistically significant difference. MW: Mann-Whitney test.
