# Supplementary material for: Operative Times, Costs and Patient‐Related Outcome Measures in Vertical Ridge Augmentation With Customised Reinforced PTFE Mesh Versus CAD/CAM Titanium Mesh: Secondary Analysis of a Randomised Clinical Trial
Source: J Clin Periodontol. 2025 May 26;52(7):971–82. doi: 10.1111/jcpe.14185 (PMC12176461; doi:10.1111/jcpe.14185)
Supplement: Supplementary file 4 — Appendix S4. Supporting Information. [file JCPE-52-971-s004.docx]

**APPENDIX S4**

**Graph A4.1**

Error bars indicated one-sided 95% confidence intervals of the difference in healing complication mean values between the Test and Control groups (Mesh minus PTFE). The broken red line delineating the difference in the score (= 0.30) shows the Δ and the data prove non-inferiority of the Ti-mesh group compared with the PTFE group. Although there is no statistically significant difference between the two treatments, the Test group tends to be worse than the Control group in terms of surgical complications.


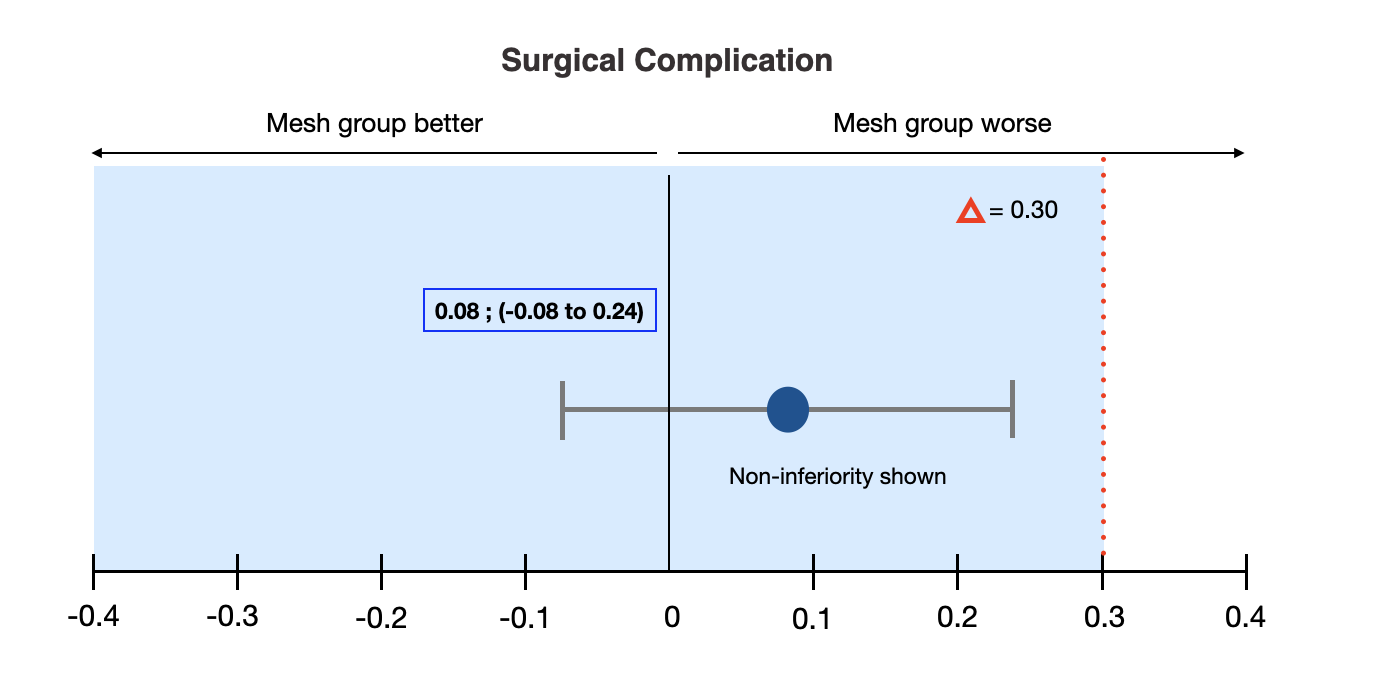


**Table A4.1**

Univariate Linear Regression Analysis of Factors Affecting Healing complication. The table summarizes the results of univariate linear regression analyses examining various factors in relation to healing outcomes. R-squared indicates the proportion of variance in healing explained by each factor. 95% CI provides the range within which the true effect is likely to fall, and p-values assess statistical significance. Notably, FANS_TOT show statistically significant relationships with healing (p < 0.05), suggesting these factors are meaningfully associated with the outcome. Other factors did not reach statistical significance.

|  | **R-squared** | **95% CI** | **p-value** |
| --- | --- | --- | --- |
| **Group**  **(Test:1 ; Control:0)** | 0.0047 | [-0.3730152, 0.596271] | 0.645 |
| **Sex**  **(F:0 ; M:1)** | 0.0638 | [-0.0503055, 0.7851892] | 0.083 |
| **Age** | 0.0115 | [-16.67061, 7.786889] | 0.468 |
| **Smoke**  **(N:0 ; Y:1)** | 0.0060 | [-0.2366263, 0.4040681] | 0.601 |
| **Quadrant** | 0.0001 | [-1.095694, 1.179414] | 0.941 |
| **Sextant** | 0.0030 | [-1.427747, 0.9812349] | 0.711 |
| **Defect_size**  **(S:1 ; M:2 ; L:3)** | 0.0112 | [-0.339892, 0.7212874] | 0.473 |
| **VBD** | 0.0072 | [-1.212403, 2.189613] | 0.566 |
| **Total_intra_op_time** | 0.0204 | [-48.1874, 16.67112] | 0.333 |
| **Anxiety_pat_t0** | 0.0046 | [-2.309567, 3.686311] | 0.646 |
| **POSSE_Tot** | 0.0202 | [-3.973016, 11.43348] | 0.335 |
| **PAIN_TOT** | 0.0178 | [-0.7763579, 2.066979] | 0.366 |
| **FANS_TOT** | 0.0992 | [0.0745336, 1.335433] | 0.029* |
| **Anxiety_op0** | 0.0001 | [-1.287042, 1.370763] | 0.950 |

** statistically significant difference*

**Table A4.2**

Univariate Linear Regression Analysis of Factors Affecting Surgical complication. The table presents the results of univariate linear regression analyses evaluating the relationship between various factors and Surg01. R-squared values indicate the proportion of variance in Surg01 explained by each factor. 95% confidence intervals (CI) and p-values are provided to assess the significance and precision of the estimates. None of the factors showed statistically significant associations with Surg01.

|  | **R-squared** | **95% CI** | **p-value** |
| --- | --- | --- | --- |
| **Group**  **(Test:1 ; Control:0)** | 0.0227 | [-0.2455126, 0.0788459] | 0.306 |
| **Sex**  **(F:0 ; M:1)** | 0.0002 | [-0.1933563, 0.1757739] | 0.924 |
| **Age** | 0.0114 | [-0.0087713, 0.0041134] | 0.470 |
| **Smoke**  **(N:0 ; Y:1)** | 0.0130 | [-0.1511728, 0.3416489] | 0.441 |
| **Quadrant** | 0.0023 | [-0.0585748, 0.0813669] | 0.745 |
| **Sextant** | 0.0000 | [-0.0660636, 0.0660636] | 1.000 |
| **Defect_size**  **(S:1 ; M:2 ; L:3)** | 0.0158 | [-0.2114731, 0.0848544] | 0.394 |
| **VBD** | 0.0016 | [-0.0529136, 0.0403748] | 0.788 |
| **Total_intra_op_time** | 0.0000 | [-0.0024811, 0.0023834] | 0.968 |
| **Anxiety_pat_t0** | 0.0191 | [-0.0139259, 0.0386082] | 0.349 |
| **POSSE_Tot** | 0.0003 | [-0.0108339, 0.0096434] | 0.907 |
| **PAIN_TOT** | 0.0126 | [-0.076244, 0.0341605] | 0.447 |
| **FANS_TOT** | 0.0516 | [-0.2086251, 0.0250533] | 0.121 |
| **Anxiety_op0** | 0.0002 | [-0.0627445, 0.0571774] | 0.926 |

**Table A4.3.**

Pre-operative, intra-operative and total times evaluated in the two study groups and overall.

| Variable | Control | | | | | Test | | | | Overall | | | | p-value | | Estimated | |
| --- | --- | --- | --- | --- | --- | --- | --- | --- | --- | --- | --- | --- | --- | --- | --- | --- | --- |
|  | Mean ± SD (Median) | | | | 95% CI | Mean ± SD (Median) | | 95% CI | | Mean ± SD (Median) | | 95% CI | |  |  | Mean (95% CI) | |
| Modeling Time | 10.25 ± 7.43 (7) | | 7.11; 13.39 | | | 0.0 ± 0 (0) | | 0; 0 | | 5.25 ± 7.30 (3.5) | | 3.13; 7.37 | | 0.0000*  MW | | -10.0 (CI: -13.09; -6.9074) | |
| Planning Time | 16.38 ± 6.59 (15.5) | | 13.59; 19.16 | | | 84.37 ± 15.48 (80) | | 77.83; 90.91 | | 50.37 ± 36.32 (50.5) | | 39.83; 60.92 | | 0.0000*  MW | | 2.21 (CI: -2.07; 6.49) | |
| Shipping Time | 5 ± 0 (5) | | 5; 5 | | | 10 ± 4.27 (10) | | 8.19; 11.80 | | 7.5 ± 3.91 (5) | | 6.36; 8.64 | | 0.0000*  MW | | 5.87 (CI: 3.24; 6.75) | |
| Total Pre-operative Time (*) | | 26.63 ± 11.90 (22.0) | | 21.60; 31.65 | | | 84.37 ± 15.48 (80) | | 77.83; 90.91 | | 55.5 ± 32.22 (60) | | 46.14; 64.85 | | 0.0000* MW | | 57.75 (CI: 49.72; 65.77) |
| Site Preparation Time | | 12.58 ± 6.20 (10) | | 9.97; 15.20 | | | 12.58 ± 6.23 | | 9.95; 15.22 | | 12.58 ± 6.15 (10) | | 10.80; 14.37 | | 0.8922  MW | | 0.0 (CI: -3.61; 3.6122) |
| Bone Harvesting Time | | 15.71 ± 10.33 (10.5) | | 11.35; 20.07 | | | 17.17 ± 12.71 (11) | | 11.80; 22.53 | | 16.44 ± 11.48 (10.5) | | 13.10; 19.77 | | 0.7713  MW | | 1.46 (CI: -5.27; 8.1882) |
| Mesh Fixation Time | | 18.33 ± 7.18 | | 15.30; 21.36 | | | 16.54 ± 6.40 | | 13.84; 19.24 | | 17.44 ± 6.79 | | 15.47; 19.41 | | 0.3662  t-test | | -1.79 (CI: -5.74; 2.1599) |
| Membrane Fixation Time | | 5.46 ± 2.64 | | 4.34; 6.57 | | | 9.29 ± 3.97 | | 7.61; 10.97 | | 7.38 ± 3.86 (7) | | 6.25; 8.50 | | 0.0003*  t-test | | 3.83 (CI: 1.87; 5.7925) |
| Flap Mobilization | | 8.00 ± 3.84 (5) | | 6.38; 9.62 | | | 8.83 ± 5.00 (5.5) | | 6.72; 10.95 | | 8.42 ± 4.43 (5.5) | | 7.13; 9.70 | | 0. 6463  MW | | 0.83 (CI: -1.76; 3.4254) |
| Suturing Time | | 36.21 ± 8.69 | | 32.54; 39.88 | | | 37.79 ± 11.93 (35.5) | | 32.75; 42.83 | | 37.00 ± 10.36 (35.5) | | 33.99; 40.01 | | 0. 8358  MW | | 1.58 (CI: -4.4820; 7.6487;) |
| Actual Intra-Operative Time | | 96.29 ± 19.09 | | 88.23; 104.35 | | | 102.21 ± 27.07 (96.0) | | 90.78; 113.64 | | 99.25 ± 23.37 (96.5) | | 92.47; 106.03 | | 0.7885 MW | | 5.92 (CI: -7.69; 19.53) |
| Total Intra-operative Time | | 121.67 ± 37.26 | | 105.93; 137.40 | | | 118.17 ± 31.29 (105.0) | | 104.96; 131.38 | | 119.92 ± 34.08 (107.0) | | 110.02; 129.81 | | 0.6875 MW | | -3.50 (CI: -23.49; 16.49) |
| TOTALTIME | | 148.29 ± 41.03 | | 130.96; 165.62 | | | 221.13 ± 30.01 (215.5) | | 208.45; 233.80 | | 184.71 ± 51.18 | | 169.85; 199.57 | | 0.0000* MW | | 72.83 (CI: 51.95; 93.72) |

For each treatment group, the table presents the values of Pre-operative, Intra-operative, and Total operative times. For each variable, the mean, standard deviation (SD), median (for variables with non-normal distribution only), and 95% confidence interval (95% CI) are reported.

*Statistically significant difference. MW: Mann-Whitney test. (*) excluding the shipping time.

**Table A4.4.**

Univariate Linear Regression Analysis of Factors Associated with Total Intraoperative Time. The table summarizes the results of univariate linear regression analyses evaluating the relationship between various factors and Total_intra_op_time. R-squared values indicate how much of the variance in intraoperative time is explained by each factor. 95% confidence intervals (CI) and p-values assess the precision and significance of these relationships. Significant associations were found with Quadrant_num, Sextant_num, Defect_size, and VBD, indicating these factors are meaningfully related to intraoperative time. Other factors did not show statistically significant relationships.

|  | **R-squared** | **95% CI** | **p-value** |
| --- | --- | --- | --- |
| **Group**  **(Test:1 ; Control:0)** | 0.0629 | [-36.29457, 2.461234] | 0.086 |
| **Sex**  **(F:0 ; M:1)** | 0.0213 | [-11.19538, 33.36681] | 0.322 |
| **Age** | 0.0001 | [-0.8199427, 0.7611662] | 0.941 |
| **Smoke**  **(N:0 ; Y:1)** | 0.0096 | [-40.11809, 20.11809] | 0.507 |
| **Quadrant** | 0.2203 | [-21.06696, -5.971973] | 0.001* |
| **Sextant** | 0.1557 | [-18.1233, -3.309132] | 0.006* |
| **Defect_size**  **(S:1 ; M:2 ; L:3)** | 0.2975 | [18.21633, 48.76496] | 0.000* |
| **VBD** | 0.1178 | [1.236128, 11.93634] | 0.017* |
| **Anxiety_pat_t0** | 0.0042 | [-2.522939, 3.935643] | 0.662 |
| **POSSE_Tot** | 0.0338 | [-0.45421, 2.002196] | 0.211 |
| **PAIN_TOT** | 0.0265 | [-2.96946, 10.40701] | 0.269 |
| **FANS_TOT** | 0.0132 | [-8.882855, 20.2017] | 0.437 |
| **Anxiety_op0** | 0.0094 | [-9.673668, 4.891483] | 0.512 |

** statistically significant difference*

**Table A4.5.**

Charges analyzed in the two study groups and overall.

| Variable | Control | | Test | | Overall |  | p-value | Estimated |
| --- | --- | --- | --- | --- | --- | --- | --- | --- |
|  | Mean ± SD(Median) | 95% CI | Mean ± SD(Median) | 95% CI | Mean ± SD(Median) | 95% CI |  | Mean (95% CI) |
| Anesthesia | 4.05 ± 1.02 (3.87) | 3.62; 4.48 | 4.34 ± 1.49 (3.87) | 3.70; 4.97 | 4.19 ± 1.27 (3.87) | 3.82; 4.56 | 0.7467  MW | 0.29 ([-0.46]; [1.03]) |
| Bone Scraper | 44 ± 0 (44) | 44; 44 | 44 ± 0 (44) | 44; 44 | 44 ± 0 (44) | 44; 44 | 1.000  MW | 0 (0; 0) |
| Digital project | 121 ± 0 (121) | 121; 121 | 121 ± 0 (121) | 121; 121 | 121 ± 0 (121) | 121; 121 | 1.000  MW | 0 (0; 0) |
| 3D Stereolitographic Model & mesh replica | 100 ± 0 (100) | 100; 100 | 0 ± 0 (0) | 100; 100 | 50 ± 50.52 (50) | 35.33; 64.67 | <0.0001* MW | -100 (-100; -100) |
| Device | 322.29 ± 45.92 | 302.90; 341.68 | 467.04 ± 79.69 | 433.39; 500.69 | 394.67 ± 97.41 (375) | 366.38; 422.95 | <0.0001* t-test | 144.75 ([106.96]; [182.54]) |
| Membranes | 172.92 ± 41.78 | 155.28; 190.56 | 155.42 ± 34.39 (140) | 140.90; 169.94 | 164.17 ± 38.87 (145) | 152.88; 175.45 | 0.0001* MW | -17.5 ([-39.73]; [4.73]) |
| Biomaterial | 254.38 ± 90.76 | 216.05; 292.70 | 247.5 ± 80.69 | 213.43; 281.57 | 250.94 ± 85.02 | 226.25; 275.63 | 0.7828  t-test | -6.88 ([-56.77]; [43.02]) |
| Screws | 81.25 ± 41.94 | 63.54; 98.96 | 107.5 ± 17.51 (120) | 100.11; 114.89 | 94.38 ± 34.45 (90) | 84.37; 104.38 | 0.0106*  MW | 26.25 ([7.58]; [44.92]) |
| Tacks | 77.58 ± 38.28 | 61.42; 93.75 | 49 ± 17.02 | 41.81; 56.19 | 63.29 ± 32.67 (56) | 53.80; 72.78 | 0.0017  t-test | -28.58 ([-45.80]; [-11.37]) |
| Sutures | 22.60 ± 8.27 (22.92) | 19.11; 26.09 | 21.65 ± 8.63 (15.28) | 18.00; 25.29 | 22.12 ± 8.38 (15.28) | 19.69; 24.56 | 0.5975 MW | -0.96 ([-5.87]; [3.96]) |
| TOTAL COST | 1200.07 ± 176.81 | 1125.41; 1274.73 | 1272.44 ± 147.55 | 1210.14; 1334.75 | 1236.19 ± 165.14 | 1188.24; 1284.14 | 0.1297  t-test | 17.37 ([-77.25]; [111.99]) |

For each treatment group, the table shows the shows the costs incurred for the treatments. For each variable, the mean, standard deviation (SD), median (only for variables with non-normal distribution), 95% confidence interval (95%CI) were reported.

*Statistically significant difference. MW: Mann-Whitney test.

**Table A4.6.**

Patient's pain level values measured over the 14 days following surgery evaluated in both groups and overall

| Variable | Control | | Test | | Overall | | p-value | Estimated Difference  Mean (95% CI) |
| --- | --- | --- | --- | --- | --- | --- | --- | --- |
|  | **Mean ± SD (Median) 95% CI** | | **Mean ± SD (Median) 95% CI** | | **Mean ± SD (Median) 95% CI** | |  |  |
| Pain Day 1 | 2.75 ± 2.69 (2) | 1.61; 3.89 | 2.19 ± 2.12 (2) | 1.29; 3.08 | 2.47 ± 2.41 (2) | 1.77; 3.17 | 0.5647  MW | -0.5625 (-1.970, 0.845) |
| Pain Day 2 | 1.92 ± 2.55 (1) | 0.84; 2.99 | 1.27 ± 1.50 (1) | 0.64; 1.90 | 1.59 ± 2.10 (1) | 0.99; 2.20 | 0.5426  MW | -0.6453 (-1.861, 0.570) |
| Pain Day 3 | 2.00 ± 2.52 (1) | 0.94; 3.06 | 1.19 ± 1.51 (1) | 0.55; 1.82 | 1.59 ± 2.10 (1) | 0.99; 2.20 | 0.2184  MW | -0.8125 (-2.019, 0.394) |
| Pain Day 4 | 1.67 ± 2.26 (1) | 0.71; 2.62 | 1.10 ± 1.40 (1) | 0.51; 1.69 | 1.39 ± 1.88 (1) | 0.84; 1.93 | 0.4770  MW | -0.5625 (-1.654, 0.529) |
| Pain Day 5 | 1.63 ± 2.24 (1) | 0.68; 2.57 | 1.17 ± 1.63 (1) | 0.48; 1.85 | 1.40 ± 1.95 (1) | 0.83; 1.96 | 0.4168  MW | -0.4583 (-1.596, 0.679) |
| Pain Day 6 | 1.46 ± 2.11 (1) | 0.57; 2.35 | 1.02 ± 1.34 (0) | 0.46; 1.59 | 1.24 ± 1.76 (1) | 0.73; 1.75 | 0.4799  MW | -0.4375 (-1.463, 0.588) |
| Pain Day 7 | 1.42 ± 2.00 (1) | 0.57; 2.26 | 1.33 ± 2.25 (1) | 0.38; 2.29 | 1.38 ± 2.11 (1) | 0.76; 1.99 | 0.2629  MW | -0.0833 (-1.321, 1.154) |
| Pain Day 8 | 1.42 ± 1.86 (1) | 0.63; 2.20 | 1.29 ± 2.21 (1) | 0.36; 2.23 | 1.35 ± 2.02 (1) | 0.77; 1.94 | 0.3388  MW | -0.125 (-1.313, 1.063) |
| Pain Day 9 | 0.96 ± 1.33 (1) | 0.39; 1.52 | 1.21 ± 2.28 (0) | 0.25; 2.17 | 1.08 ± 1.85 (0) | 0.55; 1.62 | 0.6396  MW | 0.25 (-0.835, 1.335) |
| Pain Day 10 | 0.88 ± 1.19 (0.5) | 0.37; 1.38 | 1.00 ± 1.93 (0) | 0.19; 1.81 | 0.94 ± 1.59 (0) | 0.48; 1.40 | 0.6108 MW | 0.125 (-0.806, 1.056) |
| Pain Day 11 | 0.90 ± 1.14 (0.5) | 0.41; 1.38 | 0.54 ± 1.24 (0) | 0.02; 1.07 | 0.72 ± 1.19 (0) | 0.37; 1.07 | 0.0939  MW | -0.3542 (-1.047, 0.339) |
| Pain Day 12 | 0.65 ± 0.91 (0) | 0.26; 1.03 | 0.46 ± 1.13 (0) | -0.02; 0.94 | 0.55 ± 1.02 (0) | 0.26; 0.85 | 0.2089  MW | -0.1875 (-0.785, 0.410) |
| Pain Day 13 | 0.63 ± 1.35 (0) | 0.20; 1.05 | 0.46 ± 1.13 (0) | -0.02; 0.94 | 0.54 ± 1.07 (0) | 0.23; 0.85 | 0.3265  MW | -0.1667 (-0.791, 0.458) |
| Pain Day 14 | 0.46 ± 0.72 (0) | 0.15; 0.76 | 0.46 ± 1.16 (0) | -0.04; 0.96 | 0.46 ± 0.95 (0) | 0.18; 0.74 | 0.3276  MW | -0.0018 (-0.566, 0.562) |

For each treatment group, the table shows the intensity of pain perceived (10-point VAS) by patients during the 14 days of observation. For each variable, the mean, standard deviation (SD), median (only for variables with non-normal distribution), 95% confidence interval (95%CI) were reported.

MW: Mann-Whitney test.

**Table A4.7.**

Dosage of anti-inflammatory drugs (FANS) values, reported in the 14 days following surgery, evaluated in both study groups and overall.

| Variable | Control | | Test | | Overall | | p-value | Estimated Difference |
| --- | --- | --- | --- | --- | --- | --- | --- | --- |
|  | **Mean ± SD (Median) 95% CI** | | **Mean ± SD (Median) 95% CI** | | **Mean ± SD (Median) 95% CI** | |  | (Mean, 95% CI) |
| FANS  Day 1 | 2.75 ± 2.69 | 1.61; 3.89 | 2.19 ± 2.12 | 1.29; 3.08 | 2.47 ± 2.41 | 1.77; 3.17 | 0.2995  t-test | -0.17 [-0.80, 0.47] |
| FANS  Day 2 | 1.92 ± 2.55 | 0.84; 2.99 | 1.27 ± 1.50 | 0.64; 1.90 | 1.59 ± 2.10 | 0.99; 2.20 | 0.4519  t-test | -0.04 [-0.73, 0.65] |
| FANS  Day 3 | 2.00 ± 2.52 (1) | 0.94; 3.06 | 1.19 ± 1.51 | 0.55; 1.82 | 1.59 ± 2.10 (1) | 0.99; 2.20 | 0.3091 MW | 0.38 [-0.44, 1.19] |
| FANS  Day 4 | 1.67 ± 2.26 (1) | 0.71; 2.62 | 1.10 ± 1.40 | 0.51; 1.69 | 1.39 ± 1.88 | 0.84; 1.93 | 0.5547 MW | 0.13 [-0.59, 0.84] |
| FANS  Day 5 | 1.63 ± 2.24 (1) | 0.68; 2.57 | 1.17 ± 1.63 | 0.48; 1.85 | 1.40 ± 1.95 (1) | 0.83; 1.96 | 0.6986 MW | 0.04 [-0.69, 0.77] |
| FANS  Day 6 | 1.46 ± 2.11 (1) | 0.57; 2.35 | 1.02 ± 1.34 | 0.46; 1.59 | 1.24 ± 1.76 (1) | 0.73; 1.75 | 0.7388 MW | 0.00 [-0.73, 0.73] |
| FANS  Day 7 | 1.42 ± 2.00 (1) | 0.57; 2.26 | 1.33 ± 2.25 (1) | 0.38; 2.29 | 1.38 ± 2.11 (1) | 0.76; 1.99 | 0. 1991 MW | -0.38 [-0.93, 0.18] |
| FANS  Day 8 | 1.42 ± 1.86 (1) | 0.63; 2.20 | 1.29 ± 2.21 (1) | 0.36; 2.23 | 1.35 ± 2.02 (1) | 0.77; 1.94 | 0. 3509  MW | -0.33 [-0.88, 0.21] |
| FANS  Day 9 | 0.96 ± 1.33 (1) | 0.39; 1.52 | 1.21 ± 2.28 (0) | 0.25; 2.17 | 1.08 ± 1.85 (0) | 0.55; 1.62 | 0. 8105  MW | -0.13 [-0.58, 0.33] |
| FANS  Day 10 | 0.88 ± 1.19 (0.5) | 0.37; 1.38 | 1.00 ± 1.93 (0) | 0.19; 1.81 | 0.94 ± 1.59 (0) | 0.48; 1.40 | 0. 4531  MW | -0.17 [-0.54, 0.21] |
| FANS  Day 11 | 0.90 ± 1.14 (0.5) | 0.41; 1.38 | 0.54 ± 1.24 (0) | 0.02; 1.07 | 0.72 ± 1.19 (0) | 0.37; 1.07 | 0. 3903  MW | -0.21 [-0.55, 0.13] |
| FANS  Day 12 | 0.65 ± 0.91 (0) | 0.26; 1.03 | 0.46 ± 1.13 (0) | -0.02; 0.94 | 0.55 ± 1.02 (0) | 0.26; 0.85 | 0. 9570  MW | -0.04 [-0.28, 0.20] |
| FANS  Day 13 | 0.63 ± 1.35 (0) | 0.20; 1.05 | 0.46 ± 1.13 (0) | -0.02; 0.94 | 0.54 ± 1.07 (0) | 0.23; 0.85 | 0. 3504  MW | -0.17 [-0.44, 0.11] |
| FANS  Day 14 | 0.46 ± 0.72 (0) | 0.15; 0.76 | 0.46 ± 1.16 (0) | -0.04; 0.96 | 0.46 ± 0.95 (0) | 0.18; 0.74 | 0. 9314  MW | -0.08 [-0.34, 0.18] |

For each treatment group, the average number of anti-inflammatory drugs taken by the patient. For each variable, the mean, standard deviation (SD), median (only for variables with non-normal distribution), 95% confidence interval (95%CI) were reported.

MW: Mann-Whitney test.

**Table A4.8.**

Levels of limitation in daily functions in the 14 days following surgery evaluated in the two study groups and overall

| Variable | Control | | Test | | Overall | | p-value | Estimated Difference |
| --- | --- | --- | --- | --- | --- | --- | --- | --- |
|  | **Mean ± SD (Median) 95% CI** | | **Mean ± SD (Median) 95% CI** | | **Mean ± SD (Median) 95% CI** | |  | (Mean, 95% CI) |
| Limitation  Day 1 | 2.96 ± 0.81 | 2.62; 3.30 | 2.83 ± 0.87 (3) | 2.47; 3.20 | 2.90 ± 0.83 | 2.65; 3.14 | 0.5281  MW | -0.13 (-0.61, 0.36) |
| Limitation  Day 2 | 2.79 ± 0.78 (3) | 2.46; 3.12 | 2.71 ± 0.86 (3) | 2.35; 3.07 | 2.75 ± 0.81 (3) | 2.51; 2.99 | 0.5822  MW | -0.08 (-0.56, 0.39) |
| Limitation  Day 3 | 2.58 ± 0.78 (3) | 2.26; 2.91 | 2.46 ± 0.83 | 2.11; 2.81 | 2.52 ± 0.80 (3) | 2.29; 2.75 | 0.6491  MW | -0.13 (-0.59, 0.34) |
| Limitation  Day 4 | 2.50 ± 0.72 | 2.19; 2.81 | 2.42 ± 0.88 | 2.04; 2.79 | 2.46 ± 0.80 | 2.23; 2.69 | 0.7216  t-test | -0.08 (-0.55, 0.38) |
| Limitation  Day 5 | 2.50 ± 0.72 | 2.19; 2.81 | 2.38 ± 0.93 | 1.98; 2.77 | 2.44 ± 0.82 | 2.20; 2.68 | 0.6040  t-test | -0.12 (-0.61, 0.36) |
| Limitation  Day 6 | 2.38 ± 0.65 | 2.10; 2.65 | 2.21 ± 0.93 | 1.81; 2.60 | 2.29 ± 0.80 | 2.06; 2.52 | 0.7624  t-test | -0.17 (-0.63, 0.30) |
| Limitation  Day 7 | 2.33 ± 0.99 | 2.09; 2.57 | 2.13 ± 0.99 | 1.70; 2.54 | 2.23 ± 0.81 | 1.99; 2.46 | 0.8121  t-test | -0.21 (-0.68, 0.26) |
| Limitation  Day 8 | 2.21 ± 0.66 | 1.93; 2.48 | 1.96 ± 1.00 | 1.54; 2.38 | 2.08 ± 0.85 | 1.84; 2.33 | 0.3113  t-test | -0.25 (-0.74, 0.24) |
| Limitation  Day 9 | 2.21 ± 0.66 | 1.93; 2.48 | 1.88 ± 0.95 (2) | 1.47; 2.27 | 2.04 ± 0.82 | 1.80; 2.28 | 0.0766  MW | -0.33 (-0.81, 0.14) |
| Limitation  Day 10 | 2.13 ± 0.61 | 1.87; 2.38 | 1.79 ± 0.88 (2) | 1.41; 2.16 | 1.96 ± 0.77 | 1.73; 2.18 | 0. 0852  MW | -0.33 (-0.77, 0.11) |
| Limitation  Day 11 | 2.13 ± 0.61 | 1.87; 2.38 | 1.71 ± 0.91 (2) | 1.32; 2.09 | 1.92 ± 0.79 | 1.69; 2.15 | 0.0203*  MW | -0.42 (-0.87, 0.03) |
| Limitation  Day 12 | 2.00 ± 0.51 | 1.78; 2.22 | 1.67 ± 0.82 (1.5) | 1.32; 2.01 | 1.83 ± 0.69 | 1.63; 2.03 | 0.0245*  MW | -0.33 (-0.73, 0.06) |
| Limitation  Day 13 | 2.00 ± 0.51 (2) | 1.72; 2.28 | 1.63 ± 0.77 | 1.30; 1.95 | 1.81 ± 0.73 | 1.60; 2.03 | 0.0281*  MW | -0.38 (-0.79, 0.04) |
| Limitation  Day 14 | 1.92 ± 0.72 (2) | 1.61; 2.22 | 1.61 ± 0.78 | 1.27; 1.95 | 1.77 ± 0.76 | 1.54; 1.99 | 0.0945  MW | -0.31 (-0.75, 0.13) |

For each treatment group, the average value of Limitation declared by the patient. For each variable, the mean, standard deviation (SD), median (only for variables with non-normal distribution), 95% confidence interval (95%CI) were reported.

*Statistically significant difference. MW: Mann-Whitney test.

**Table A4.9.**

Post-operative symptoms evaluated in both study groups: frequency distribution and numerical categorization analysis

| Variable | Control  (No / Yes) | Test  (No / Yes) | p-value | Control  (Mean ± SD, Median) | Test  (Mean ± SD, Median) | Overall  (Mean ± SD, Median) | Mann-Whitney p-value |
| --- | --- | --- | --- | --- | --- | --- | --- |
| Swelling  Day 1 | No 0.00%  Yes 100.00% | No 0.00%  Yes 100.00% | N/A | 1.00 ± 0.00 (1) | 1.00 ± 0.00 (1) | 1.00 ± 0.00 (1) | 1.0000 |
| Swelling  Day 3 | No 8.33%  Yes 91.67% | No 25.00%  Yes 75.00% | 0.245 FE | 1.00 ± 0.28 (1) | 0.92 ± 0.28 (1) | 0.96 ± 0.20 (1) | 0.4894 |
| Swelling  Day 7 | No 4.00%  Yes 91.67% | No 25.00%  Yes 75.00% | 0.122 FE | 0.92 ± 0.44 (1) | 0.75 ± 0.44 (1) | 0.83 ± 0.38 (1) | 0.2448 |
| Swelling  Day 14 | No 37.50%  Yes 62.50% | No 58.33%  Yes 41.67% | 0.149 Pχ² | 0.62 ± 0.49 (1) | 0.42 ± 0.50 (0) | 0.52 ± 0.50 (1) | 0.2476 |
| Nausea Day 1 | No 100.00%  Yes 0.00% | No 95.83%  Yes 4.17% | 0.500 FE | 0.00 ± 0.00 (0) | 0.04 ± 0.20 (0) | 0.02 ± 0.14 (0) | 1.0000 |
| Nausea Day 3 | No 100.00%  Yes 0.00% | No 100.00%  Yes 0.00% | N/A | 0.00 ± 0.00 (0) | 0.00 ± 0.00 (0) | 0.00 ± 0.00 (0) | 1.0000 |
| Nausea Day 7 | No 100.00%  Yes 0.00% | No 100.00%  Yes 0.00% | N/A | 0.00 ± 0.00 (0) | 0.00 ± 0.00 (0) | 0.00 ± 0.00 (0) | 1.0000 |
| Nausea Day 14 | No 100.00%  Yes 0.00% | No 95.83%  Yes 4.17% | 0.500 FE | 0.00 ± 0.00 (0) | 0.04 ± 0.20 (0) | 0.02 ± 0.14 (0) | 1.0000 |
| Bruising  Day 1 | No 70.83%  Yes 29.17% | No 66.67%  Yes 33.33% | 0.755 Pχ² | 0.29 ± 0.46 (0) | 0.33 ± 0.48 (0) | 0.31 ± 0.47 (0) | 1.0000 |
| Bruising  Day 3 | No 41.67%  Yes 58.33% | No 33.33%  Yes 66.67% | 0.551 Pχ² | 0.58 ± 0.50 (1) | 0.67 ± 0.48 (1) | 0.62 ± 0.49 (1) | 0.7661 |
| Bruising  Day 7 | No 45.83%  Yes 54.17% | No 45.83%  Yes 54.17% | 1.000 Pχ² | 0.54 ± 0.50 (1) | 0.54 ± 0.51 (1) | 0.54 ± 0.50 (1) | 1.0000 |
| Bruising  Day 14 | No 66.67%  Yes 33.33% | No 87.50%  Yes 12.50% | 0.086 Pχ² | 0.33 ± 0.48 (0) | 0.13 ± 0.34 (0) | 0.23 ± 0.42 (0) | 0.1681 |
| Bleeding  Day 1 | No 91.67%  Yes 8.33% | No 100.00%  Yes 0.00% | 0.245 FE | 0.08 ± 0.28 (0) | 0.04 ± 0.20 (0) | 0.04 ± 0.20 (0) | 0.4894 |
| Bleeding  Day 3 | No 100.00%  Yes 0.00% | No 100.00%  Yes 0.00% | N/A | 0.00 ± 0.00 (0) | 0.00 ± 0.00 (0) | 0.00 ± 0.00 (0) | 1.0000 |
| Bleeding  Day 7 | No 100.00%  Yes 0.00% | No 95.83%  Yes 4.17% | 0.500 FE | 0.00 ± 0.00 (0) | 0.04 ± 0.20 (0) | 0.02 ± 0.14 (0) | 1.0000 |
| Bleeding  Day 14 | No 100.00%  Yes 0.00% | No 100.00%  Yes 0.00% | N/A | 0.00 ± 0.00 (0) | 0.00 ± 0.00 (0) | 0.00 ± 0.00 (0) | 1.0000 |
| Fever Day 1 | No 100.00%  Yes 0.00% | No 95.83%  Yes 4.17% | 0.500 FE | 0.04 ± 0.20 (0) | 0.04 ± 0.20 (0) | 0.04 ± 0.20 (0) | 1.0000 |
| Fever Day 3 | No 95.83%  Yes 4.17% | No 95.83%  Yes 4.17% | 0.755 FE | 0.04 ± 0.20 (0) | 0.04 ± 0.20 (0) | 0.04 ± 0.20 (0) | 1.0000 |
| Fever Day 7 | No 100.00%  Yes 0.00% | No 100.00%  Yes 0.00% | N/A | 0.00 ± 0.00 (0) | 0.00 ± 0.00 (0) | 0.00 ± 0.00 (0) | 1.0000 |
| Fever Day 14 | No 100.00%  Yes 0.00% | No 100.00%  Yes 0.00% | N/A | 0.00 ± 0.00 (0) | 0.00 ± 0.00 (0) | 0.00 ± 0.00 (0) | 1.0000 |
| Lack of Sensitivity  Day 1 | No 70.83%  Yes 29.17% | No 66.67%  Yes 33.33% | 0.755 Pχ² | 0.29 ± 0.46 (0) | 0.33 ± 0.48 (0) | 0.31 ± 0.47 (0) | 1.0000 |
| Lack of Sensitivity  Day 3 | No 75.00%  Yes 25.00% | No 66.67%  Yes 33.33% | 0.525 Pχ² | 0.25 ± 0.44 (0) | 0.33 ± 0.48 (0) | 0.29 ± 0.46 (0) | 0.7516 |
| Lack of Sensitivity  Day 7 | No 83.33%  Yes 16.67% | No 83.33%  Yes 16.67% | 0.650 FE | 0.17 ± 0.38 (0) | 0.17 ± 0.38 (0) | 0.17 ± 0.38 (0) | 1.0000 |
| Lack of Sensitivity  Day 14 | No 83.33%  Yes 16.67% | No 83.33%  Yes 16.67% | 0.650 FE | 0.17 ± 0.38 (0) | 0.17 ± 0.38 (0) | 0.17 ± 0.38 (0) | 1.0000 |
| Opening Difficulties  Day 1 | No 20.83%  Yes 79.17% | No 45.83%  Yes 54.17% | 0.066 Pχ² | 0.79 ± 0.41 (1) | 0.54 ± 0.51 (1) | 0.67 ± 0.48 (1) | 0.1246 |
| Opening Difficulties  Day 3 | No 33.33%  Yes 66.67% | No 50.00%  Yes 50.00% | 0.242 Pχ² | 0.67 ± 0.48 (1) | 0.50 ± 0.51 (0.5) | 0.58 ± 0.49 (1) | 0.3801 |
| Opening Difficulties  Day 7 | No 37.50%  Yes 62.50% | No 66.67%  Yes 33.33% | 0.043* Pχ² | 0.63 ± 0.49 (1) | 0.33 ± 0.48 (0) | 0.48 ± 0.50 (1) | 0.0820 |
| Opening Difficulties  Day 14 | No 58.33%  Yes 41.67% | No 79.17%  Yes 20.83% | 0.119 Pχ² | 0.42 ± 0.50 (0) | 0.21 ± 0.41 (0) | 0.31 ± 0.47 (0) | 0.2124 |

The table presents the distribution values (as a binomial categorical variable) and the mean values (as a numeric categorical variable) of postoperative symptoms reported by patients in the two treatment groups. For each variable, the mean, standard deviation (SD), median (for variables with non-normal distribution only), and 95% confidence interval (95% CI) are reported.

N/A: Not Applicable. FE: Fischer’s exact test. Pχ²: Pearson Chi-square test. * Statistically significant difference.

**Table A4.10.**

Willingness to undergo the same surgery again evaluated in the two study groups: frequency distribution and numerical categorization analysis

| Variable | Control | Test | p-value (Fischer’s exact test) | Control  (Mean ± SD, Median) | Test  (Mean ± SD, Median) | Overall  (Mean ± SD, Median) | Mann-Whitney  p-value |
| --- | --- | --- | --- | --- | --- | --- | --- |
| Repetition Day 7 | 1: 58.33%  2: 20.83%  3: 16.67%  4: 4.17% | 1: 70.83%  2: 8.33%  3: 16.67%  4: 4.17% | 0.771 | 1.67 ± 0.92 (1) | 1.54 ± 0.93 (1) | 1.60 ± 0.92 (1) | 0.5292 |
| Repetition Day 14 | 1: 62.50%  2: 12.50%  3: 20.83%  4: 4.17% | 1: 75.00%  2: 12.50%  3: 4.17%  4: 8.33% | 0.466 | 1.67 ± 0.96 (1) | 1.46 ± 0.93 (1) | 1.56 ± 0.94 (1) | 0.3723 |

The table presents the distribution values (as a 4-category variable) and the mean values (as a numeric categorical variable) for willingness to undergo the same surgery again in the two treatment groups. For each variable, the mean, standard deviation (SD), median (for variables with non-normal distribution only), and 95% confidence interval (95% CI) are reported.

**Table A4.11.**

Univariate Linear Regression Analysis of Factors Associated with “Willingness to undergo to the same surgery”. The table presents the results of univariate linear regression analyses evaluating the relationship between various factors and REP_tot. R-squared values indicate the proportion of variance in REP_tot explained by each factor. 95% confidence intervals (CI) and p-values are provided to assess the significance and precision of the estimates. Some factors, including PAIN_TOT and Anxiety_pat_t0, showed statistically significant associations with REP_tot.

|  | **R-squared** | **95% CI** | **p-value** |
| --- | --- | --- | --- |
| **Group**  **(Test:1 ; Control:0)** | 0.0135 | -0.321 to 0.737 | 0.4321 |
| **Sex**  **(F:0 ; M:1)** | 0.0005 | -0.555 to 0.643 | 0.8833 |
| **Age** | 0.0226 | -0.031 to 0.010 | 0.3082 |
| **Smoke**  **(N:0 ; Y:1)** | 0.0049 | -0.994 to 0.613 | 0.6355 |
| **Quadrant** | 0.0497 | -0.393 to 0.051 | 0.1276 |
| **Sextant** | 0.0546 | -0.378 to 0.040 | 0.1099 |
| **Defect_size**  **(S:1 ; M:2 ; L:3)** | 0.0170 | -0.268 to 0.694 | 0.3773 |
| **VBD** | 0.0017 | -0.173 to 0.130 | 0.7786 |
| **Total_intra_op_time** | 0.0198 | -0.004 to 0.012 | 0.3404 |
| **Anxiety_pat_t0** | 0.1253 | 0.022 to 0.183 | 0.0136* |
| **POSSE_Tot** | 0.0160 | -0.019 to 0.047 | 0.3914 |
| **PAIN_TOT** | 0.1600 | 0.078 to 0.408 | 0.0049* |
| **FANS_TOT** | 0.0166 | -0.217 to 0.556 | 0.3825 |
| **Anxiety_op0** | 0.0173 | -0.279 to 0.107 | 0.3729 |

** statistically significant difference*

**Table A4.12.**

Oral Health Impact Profile-14 (OHIP-14) questionnaire and Postoperative Symptom Severity Scale (PoSSe) evaluated in the two study groups and overall.

| Variable | Control  (Mean ± SD) 95% CI | | Test  (Mean ± SD) 95% CI | | Overall  (Mean ± SD) 95% CI | | p-value | Estimated Difference  (Mean, 95% CI) |
| --- | --- | --- | --- | --- | --- | --- | --- | --- |
| POSSE_TOT | 35.67 ± 8.80 | 31.95; 39.38 | 33.25 ± 7.31 | 30.16; 36.34 | 34.46 ± 8.10 | 32.11; 36.81 | 0.3061  t-test | -2.42 (-7.12, 2.28) |
| OHIP_TOT | 11.46 ± 8.52 | 7.86; 15.05 | 14.17 ± 13.55 (9) | 8.44; 19.89 (9) | 12.81 ± 11.28 (10.5) | 9.54; 16.09 (10.5) | 0.6637  MW | 2.71 (-3.87, 9.29) |

POSSE and OHIP questionnaire scores for each treatment group at the end of follow-up. For each variable, the mean, standard deviation (SD), median (only for variables with non-normal distribution), 95% confidence interval (95%CI) were reported.

MW: Mann-Whitney test.

**Table A4.13.**

Univariate Linear Regression Analysis of Factors Associated with POSSE_Tot. The table presents the results of univariate linear regression analyses examining the relationship between various factors and POSSE_Tot. The R-squared values reflect the proportion of variance in POSSE_Tot explained by each factor. Significant associations were observed with PAIN_TOT and FANS_TOT, suggesting these variables are meaningfully related to POSSE_Tot. Other factors did not show statistically significant relationships, as indicated by their higher p-values.

|  | **R-squared** | **95% CI** | **p-value** |
| --- | --- | --- | --- |
| **Group**  **(Test:1 ; Control:0)** | 0.0053 | [-5.908721, 3.575388] | 0.623 |
| **Sex**  **(F:0 ; M:1)** | 0.0017 | [-4.602265, 6.087979] | 0.781 |
| **Age** | 0.0001 | [-1.927907, 1.827707] | 0.957 |
| **Smoke**  **(N:0 ; Y:1)** | 0.0279 | [-3.039695, 11.13493] | 0.256 |
| **Quadrant** | 0.0029 | [-2.397681, 1.65694] | 0.715 |
| **Sextant** | 0.0010 | [-2.116436, 1.711031] | 0.832 |
| **Defect_size**  **(S:1 ; M:2 ; L:3)** | 0.0453 | [-1.124257, 7.334329] | 0.146 |
| **VBD** | 0.0033 | [-1.088635, 1.612759] | 0.698 |
| **Total_intra_op_time** | 0.0338 | [-0.025653, 0.112967] | 0.211 |
| **Anxiety_pat_t0** | 0.0301 | [-2.522939, 3.935643] | 0.239 |
| **PAIN_TOT** | 0.2576 | [1.366276, 4.140851] | 0.000* |
| **FANS_TOT** | 0.1428 | [1.208525, 7.646956] | 0.008* |
| **Anxiety_op0** | 0.0092 | [-2.290875, 1.169093] | 0.517 |

** statistically significant difference*

**Table A4.14.**

Univariate Linear Regression Analysis of Factors Associated with PAIN_TOT. The table presents the results of univariate linear regression analyses investigating the impact of various factors on total postoperative pain (PAIN_TOT). The R-squared values indicate how much of the variance in PAIN_TOT is explained by each predictor. The 95% Confidence Intervals (CI) reflect the range in which the true effect is likely to be found, while p-values assess the statistical significance. Significant findings were observed for POSSE_Tot, Anxiety_pat_t0, FANS_1w and FANS_2w (p < 0.05), suggesting that these factors are strongly associated with overall pain after surgery. The high R-squared values for these variables also imply that they explain a substantial portion of the variance in total postoperative pain. Other factors, however, did not achieve statistical significance, indicating a weaker or negligible association with the outcome.

|  | **R-squared** | **95% CI** | **p-value** |
| --- | --- | --- | --- |
| **Group**  **(Test:1 ; Control:0)** | 0.0003 | -0.8225 to 0.9301 | 0.902 |
| **Sex**  **(F:0 ; M:1)** | 0.0029 | -0.8052 to 1.1641 | 0.715 |
| **Age** | 0.0208 | -0.0511 to 0.0174 | 0.328 |
| **Smoke**  **(N:0 ; Y:1)** | 0.0203 | -0.6753 to 1.9477 | 0.334 |
| **Quadrant** | 0.0464 | -0.6370 to 0.0940 | 0.142 |
| **Sextant** | 0.0073 | -0.4531 to 0.2502 | 0.564 |
| **Defect_size**  **(S:1 ; M:2 ; L:3)** | 0.0625 | -0.1005 to 1.4446 | 0.087 |
| **VBD** | 0.0538 | -0.0476 to 0.4376 | 0.113 |
| **Total_intra_op_time** | 0.0265 | -0.0057 to 0.0199 | 0.269 |
| **Anxiety_pat_t0** | 0.1354 | 0.0439 to 0.3074 | 0.010* |
| **POSSE_Tot** | 0.2576 | 0.0464 to 0.1407 | 0.0002* |
| **FANS_1w** | 0.1737 | 0.2113 to 0.9873 | 0.003* |
| **FANS_2w** | 0.1371 | 0.2729 to 1.8651 | 0.010* |
| **Anxiety_op0** | 0.0362 | -0.5198 to 0.1092 | 0.195 |

** statistically significant difference*

**Table A4.15.**

Univariate Linear Regression Analysis of Factors Associated with FANS_TOT. The table presents the results of univariate linear regression analyses evaluating the relationship between various factors and the total number of anti-inflammatory drugs taken by the patient (FANS_TOT). R-squared values indicate the proportion of variance in FANS_TOT explained by each factor. 95% confidence intervals (CI) and p-values are provided to assess the significance and precision of the estimates. Some factors, including PAIN_TOT and POSSE_TOT, showed statistically significant associations with FANS_TOT.

|  | **R-squared** | **95% CI** | **p-value** |
| --- | --- | --- | --- |
| **Group**  **(Test:1 ; Control:0)** | 0.1964 | [-0.2052, 0.5981] | 0.330 |
| **Sex**  **(F:0 ; M:1)** | -0.0336 | [-0.4901, 0.4229] | 0.883 |
| **Age** | -0.0116 | [-0.0273, 0.0041] | 0.143 |
| **Smoke**  **(N:0 ; Y:1)** | 0.1667 | [-0.4449, 0.7782] | 0.586 |
| **Quadrant** | -0.0889 | [-0.2601, 0.0824] | 0.302 |
| **Sextant** | -0.0907 | [-0.2519, 0.0705] | 0.263 |
| **Defect_size**  **(S:1 ; M:2 ; L:3)** | 0.1749 | [-0.1909, 0.5407] | 0.341 |
| **VBD** | 0.0291 | [-0.0861, 0.1442] | 0.614 |
| **Total_intra_op_time** | 0.0023 | [-0.0037, 0.0083] | 0.437 |
| **Anxiety_pat_t0** | 0.0178 | [-0.0476, 0.0832] | 0.587 |
| **POSSE_Tot** | 0.0323 | [0.0088, 0.0557] | 0.008* |
| **PAIN_TOT** | 0.2090 | [0.0864, 0.3316] | 0.001* |
| **Anxiety_op0** | -0.0985 | [-0.2440, 0.0469] | 0.179 |

** statistically significant difference*

**Graph A4.2.**


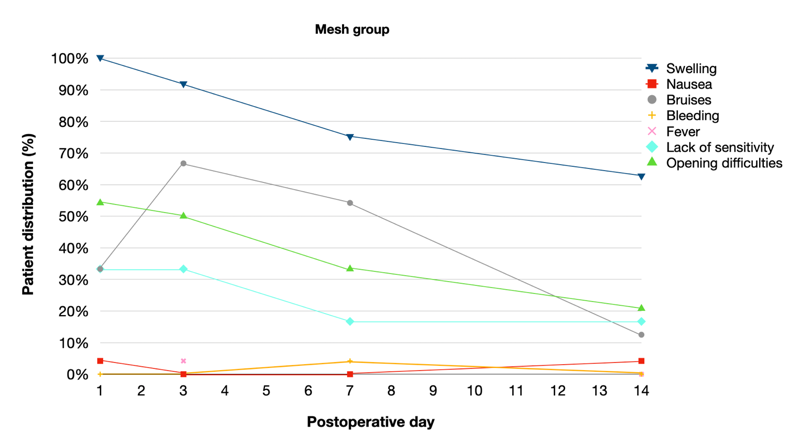

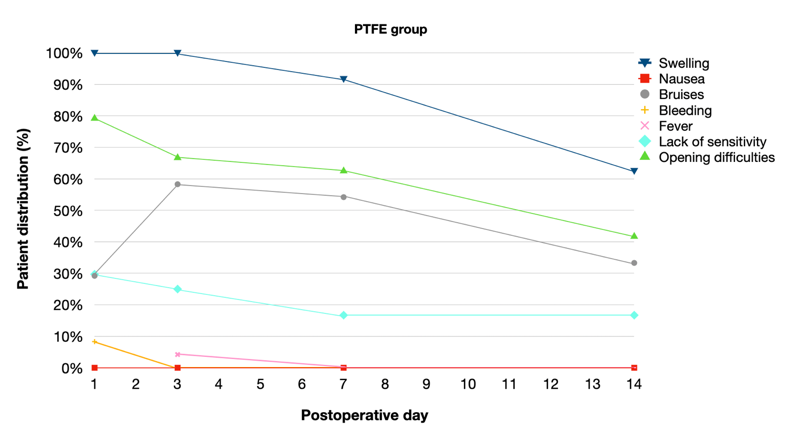
Patient distribution according to the incidence of post-operative symptoms (swelling, nausea, bruises, bleeding, fever, lack of sensitivity, opening difficulties) entered in a daily diary at days 1, 3, 7, and 14 after surgery in the PTFE group and Mesh group.
